# Triglyceride–Glucose-Based Anthropometric Indices for Predicting Incident Cardiovascular Disease: Relative Fat Mass (RFM) as a Robust Indicator

**DOI:** 10.3390/nu17132212

**Published:** 2025-07-03

**Authors:** Xinlei Chu, Haozhi Niu, Ning Wang, Yu Wang, Hongkai Xu, Huiying Wang, Liting Wu, Wei Li, Lei Han

**Affiliations:** 1 School of Public Health, Nanjing Medical University, Nanjing 211166, China; 2 Key Laboratory for Disease Prevention and Control and Health Promotion of Shaanxi Province, Department of Epidemiology and Biostatistics, School of Public Health, Global Health Institute, Xi’an Jiaotong University Health Science Center, Xi’an 710061, China; 3 Key Laboratory of Environmental Medicine Engineering, Ministry of Education, School of Public Health, Southeast University, Nanjing 210009, China; 4 Department of Occupational Disease Prevention, Jiangsu Provincial Center for Disease Control and Prevention, Nanjing 210009, China

**Keywords:** cardiovascular disease (CVD), triglyceride–glucose (TyG), anthropometric indicator (AI)

## Abstract

**Background/Objectives**: The triglyceride–glucose (TyG) index is a recognized marker for cardiovascular disease (CVD) risk linked to insulin resistance. Combining TyG with anthropometric indicators (AIs) may improve risk prediction, but the comparative value of different AIs, including novel ones like Relative Fat Mass (RFM), is unclear. This study aimed to identify which combination of TyG and AIs has the strongest association with incident CVD in a middle-aged and elderly Chinese cohort. **Methods**: In this prospective study, we evaluated the association between the cumulative average of TyG combined with eight AIs (TyG-AIs) and incident CVD, heart disease, and stroke. Using data from 5192 participants in the China Health and Retirement Longitudinal Study (CHARLS), we used multivariable logistic regression to compare the predictive value of these composite indices. **Results**: During follow-up, 1382 (26.6%) participants developed CVD. After full adjustment, the TyG index alone was only significantly associated with stroke. In contrast, most TyG-AIs showed stronger associations with all outcomes. Notably, the index combining TyG with Relative Fat Mass (TyG-RFM) exhibited the most robust associations with total CVD (OR = 2.236), heart disease (OR = 1.679), and stroke (OR = 3.288) when comparing the highest to lowest quartiles. **Conclusions**: Cumulative average TyG-AIs, particularly TyG-RFM, demonstrated more robust associations with incident CVD than the TyG index alone. The TyG-RFM index shows promise as a valuable tool to improve cardiovascular risk stratification, especially for identifying at-risk non-obese individuals.

## 1. Introduction

Cardiovascular disease (CVD) remains a leading health issue worldwide. CVD is responsible for 18 million deaths and 366 million disability-adjusted life years (DALYs) lost each year [1]. This escalating burden underscores the profound strain on healthcare systems worldwide [2]. Thus, identifying modifiable CVD risk factors is critical for formulating effective early prevention strategies [1,3].

Insulin resistance (IR) functions as a key pathophysiological mechanism responsible for multiple metabolic abnormalities, for instance, type 2 diabetes mellitus (T2DM), metabolic syndrome, and nonalcoholic fatty liver disease (NAFLD) [4]. Accumulating evidence suggests that IR not only contributes to dysregulated glucose and lipid metabolism stemming from reduced cellular sensitivity to insulin but is also a major contributor to CVD risk [5]. Derived from levels of fasting triglycerides and glucose, IR can be indirectly assessed using the triglyceride–glucose (TyG) index. This index is broadly accepted as a validated tool for gauging IR and predicting adverse health outcomes [6]. This association has been robustly confirmed through prospective, large-scale cohort studies [7]. Furthermore, obesity, a major global health challenge, exacerbates metabolic disturbances, particularly glucose intolerance and IR [8,9]. Notably, the synergistic application of the TyG index with conventional anthropometric indicators (AIs) demonstrates stronger associations with CVD incidence than TyG alone [10,11]. These integrated indices (triglyceride–glucose-anthropometric indicators, TyG-AIs) thus offer superior cardiovascular risk stratification, especially within the context of obesity-associated metabolic dysregulation.

Nonetheless, prior research has concentrated on a restricted set of AIs, including body mass index (BMI), waist circumference (WC), and waist-to-height ratio (WHtR) [12,13,14]. These conventional AIs are recognized as having limitations, particularly in accurately reflecting fat distribution and central obesity. In subjects classified with normal BMI, those with central obesity still exhibit an increased risk of CVD [15]. Emerging AIs encompass measures like the weight-adjusted waist index (WWI), a body shape index (ABSI), body roundness index (BRI), conicity index (CI), alongside relative fat mass (RFM) [16,17,18,19]. Developed to better estimate whole-body fat percentage than the traditional BMI, RFM has demonstrated stronger associations with a range of cardiometabolic outcomes in recent large-scale studies. For instance, it was reported to be a superior predictor of type 2 diabetes, heart failure, and diabetes-related mortality compared to conventional anthropometric measures [20,21,22].Nevertheless, while the synergy between TyG and conventional AIs is increasingly recognized, comprehensive, head-to-head comparisons are scarce, and the potential of novel AIs in this context remains largely unexplored. It is currently unclear which, if any, of the numerous available TyG-AI combinations offers the most robust association with cardiovascular outcomes.

This study aimed to systematically compare eight distinct TyG-AI indices—encompassing both conventional and novel markers—within a large, prospective nationwide cohort (CHARLS). Our primary objective was to identify the specific TyG-AI combination that exhibits the strongest association with incident total CVD, heart disease, and stroke, thereby providing clear evidence to optimize risk stratification strategies.

## 2. Materials and Methods

### 2.1. Study Design and Data Collection

#### 2.1.1. Study Design

This research employed data from CHARLS, a longitudinal cohort study representative of the national population, focusing on adults 45 years of age or older residing in mainland China. With its focus on adults aged 45 and older, large sample size, and comprehensive collection of biomarker data, CHARLS provides an ideal setting to investigate predictors of incident cardiovascular disease. Data collection at baseline commenced in 2011, and participants were followed up biennially in subsequent surveys. The study protocol obtained approval from the Biomedical Ethics Review Committee of Peking University. Furthermore, securing written informed consent from all individuals was a prerequisite for enrollment. Comprehensive descriptions of the CHARLS design and methodology were published in earlier works [23].

**Figure 1:**
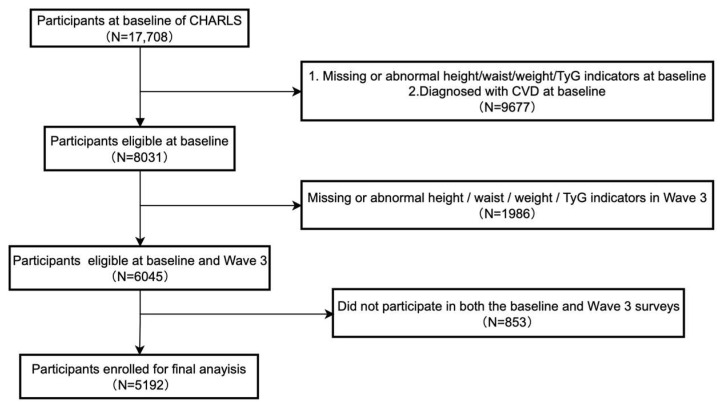
Flowchart of the study population inclusion.

For this research, individuals were considered eligible if they satisfied all of the following criteria for inclusion: (1) had participated in the baseline survey (Wave 1) and in Wave 3; (2) did not have a previous diagnosis of cardiovascular disease at baseline; and (3) provided complete data necessary for calculating the TyG index, along with measurements for height, WC, and weight, during both Wave 1 and Wave 3. Individuals were excluded if they were younger than 45 years at baseline, had incomplete data regarding their baseline CVD status, or exhibited TyG index components or anthropometric indicators that fell outside the established valid ranges at either wave of the survey. Implementation of these inclusion/exclusion rules yielded a final analytical group of 5,192 participants (Figure 1).

#### 2.1.2. Data Collection

Data collected for this research encompassed several domains. Demographic information included sex, age, educational attainment, household registration (Hukou), and marital status. Anthropometric and physiological parameters measured were systolic blood pressure (SBP), diastolic blood pressure (DBP), and BMI. Data on lifestyle habits covered smoking status and alcohol consumption status. Information regarding past medical diagnoses included existing health conditions (dyslipidemia, hypertension, diabetes mellitus, kidney issues, and liver disorders) along with the usage of pertinent medications (lipid-lowering agents, antihypertensive medications, and glucose-lowering treatments). Laboratory tests provided values for glycated hemoglobin (HbA1c), fasting blood glucose (FBG), triglycerides (TG), total cholesterol (TC), high-density lipoprotein cholesterol (HDL-c), and low-density lipoprotein cholesterol (LDL-c).

### 2.2. Data Assessment and Definitions

#### 2.2.1. Exposure Assessment

Within this research, TyG served to evaluate IR. TyG-AIs were derived by multiplying TyG with eight distinct AIs [10]. The primary exposure variable was defined as the cumulative average value of these TyG-AIs calculated between the baseline survey and Wave 3. The specific formulas used for TyG and the AIs are detailed below [12,13,14,16,17,18,19]:

$$\mathrm{TyG}=\ln\left(\frac{\mathrm{TG}\;(\mathrm{mg}/\mathrm{dL})\times \mathrm{FPG}\;(\mathrm{mg}/\mathrm{dL})}{2}\right)$$

$$\mathrm{BMI}=\frac{\mathrm{Weight}\;(\mathrm{kg})}{{\mathrm{Height}\;\left(m\right)}^{2}}$$

$$\mathrm{WHtR}=\frac{\mathrm{Waist}\;\mathrm{Circumference}\;(\mathrm{cm})}{\mathrm{Height}\;(\mathrm{cm})}$$

$$A\;\mathrm{Body}\;\mathrm{Shape}\;\mathrm{Index}\;(\mathrm{ABSI})=\frac{\mathrm{Waist}\;\mathrm{Circumference}\;(m)}{{\mathrm{BMI}}^{2/3}\times {\mathrm{Height}\;\left(m\right)}^{1/2}}$$

$$\mathrm{Weight}-\mathrm{Adjusted}\;\mathrm{Waist}\;\mathrm{Index}\;(\mathrm{WWI})=\frac{\mathrm{Waist}\;\mathrm{Circumference}\;(\mathrm{cm})}{{\mathrm{Weight}\left(\mathrm{kg}\right)}^{1/2}}$$

$$\mathrm{Conicity}\;\mathrm{Index}\;(\mathrm{CI})=\frac{\mathrm{Waist}\;\mathrm{Circumference}\;(m)}{0.109\times \sqrt{\mathrm{Weight}\;(\mathrm{kg})/\mathrm{Height}\;(m)}}$$

$$\mathrm{Body}\;\mathrm{Roundness}\;\mathrm{Index}\;(\mathrm{BRI})=364.2-365.5\times \sqrt{1-{\left(\frac{\mathrm{Waist}\;\mathrm{Circumference}\;(m)/\left(2\pi \right)}{0.5\times \mathrm{Height}\;(m)}\right)}^{2}}$$

$$\mathrm{Relative}\;\mathrm{Fat}\;\mathrm{Mass}\;(\mathrm{RFM})-\mathrm{Male}=64-\left(20\times \frac{\mathrm{Height}\;(m)}{\mathrm{Waist}\;\mathrm{Circumference}\;(m)}\right)$$

$$\mathrm{Relative}\;\mathrm{Fat}\;\mathrm{Mass}\;(\mathrm{RFM})-\mathrm{Female}=76-\left(20\times \frac{\mathrm{Height}\;(m)}{\mathrm{Waist}\;\mathrm{Circumference}\;(m)}\right)$$

$$\mathrm{Cumulative}\;\mathrm{Average}\;\mathrm{TyG}-\mathrm{AI}=\frac{\left({\mathrm{TyG}-\mathrm{AI}}_{2011}+{\mathrm{TyG}-\mathrm{AI}}_{2015}\right)}{2}$$

#### 2.2.2. Definition of CVD

Incident CVD served as the principal outcome indicator for this research. During every round of the CHARLS survey, individuals were queried about their health status. Specifically, they were asked if a doctor had ever diagnosed them with stroke or heart disease (encompassing conditions like heart attack, coronary heart disease, angina pectoris, congestive heart failure, or other cardiac issues). Additionally, they were questioned about currently receiving any treatments (including traditional Chinese medicine, Western medicine, other therapies, or none) specifically for stroke/heart disease or related complications, as part of the survey administration. In line with prior research, individuals who reported either a physician’s diagnosis of heart disease/stroke or indicated receiving targeted treatment for these conditions were categorized as having CVD [24,25,26].

#### 2.2.3. Definition of Hypertension

In this research, baseline hypertension status was assessed through participant self-reports and SBP and DBP readings. Hypertension was established if participants met one or both of these conditions: (1) participant confirmation of a prior hypertension diagnosis or the utilization of antihypertensive therapy at baseline; (2) SBP ≥ 140 mmHg or DBP ≥ 90 mmHg [27].

#### 2.2.4. Definition of Diabetes

In this research, baseline diabetes status was evaluated using participants’ self-reports alongside FBG and HbA1c measurements. Diabetes was characterized by fulfilling at least one of these criteria: (1) reporting a previous diagnosis of diabetes or current treatment with antidiabetic drugs; (2) FBG levels ≥ 7.0 mmol/L, or HbA1c levels ≥ 6.5% [28].

#### 2.2.5. Definition of Obesity

In this research, obesity was defined on the basis of recent diagnostic criteria [29]. Participants were categorized as obese if they satisfied a minimum of two out of three specified criteria: (1) a BMI greater than 23.9 kg/m^2^; (2) a WC exceeding 90 cm for male or 85 cm for female; (3) a WHtR greater than 0.5. Alternatively, BMI exceeding 30 kg/m^2^ was also considered obesity.

### 2.3. Statistical Analyses

Baseline characteristics of participants were compared as follows: continuous data following a normal distribution were reported as mean ± SD and compared using independent sample *t*-test, whereas data not normally distributed were presented as median (IQR) and evaluated with the Mann–Whitney U test; counts (percentages) were used for categorical data, and differences were assessed using Pearson’s χ^2^ test.

Spearman’s rank correlation was used to explore the correlations among the different AIs themselves (Table S1), and to assess the correlation between AIs and metabolic parameters (Figure S1). Based on cumulative average TyG-AI values, individuals were divided into four groups (Q1–Q4, Q1 was set as the reference group). To evaluate the association of cumulative average TyG-AIs with incident CVD, multivariable logistic regression analyses were conducted, yielding findings expressed as odds ratios (ORs) with 95% confidence intervals (CIs). Three sequential models were established: Model 1 (crude); Model 2 (controlling for age and sex); and Model 3 (incorporating additional adjustments for smoking status, alcohol consumption status, marital status, educational attainment, diabetes, and hypertension). Additionally, we performed logistic regression analyses for each individual AI with incident CVD in Model 3. Due to low covariate missingness (missing rate < 1.5%; Supplementary Figure S2), analyses were restricted to complete cases [30]. Multicollinearity was assessed using standardized generalized variance inflation factors (GVIFs); all standardized GVIF values were <3 (Supplementary Table S2), indicating the absence of severe multicollinearity [31]. The final multivariable models were assessed for goodness-of-fit and calibration, and all were found to be acceptable. The study investigated nonlinear relationships through the use of restricted cubic splines (RCSs) within a fully adjusted model. To formally compare the predictive discrimination of different indices, the areas under the receiver operating characteristic curve (AUCs) were calculated and compared. We used DeLong’s test to assess the statistical significance of the differences between AUCs derived from the same subjects. TyG-RFM was prespecified as the reference for all comparisons. To explore potential effect modification, we conducted several exploratory subgroup analyses, categorizing participants by obesity status, sex, and age groups (<55 versus ≥55 years). R software (version 4.4.3) was employed for all analyses, using two-sided tests with a significance level established at α = 0.05.

## 3. Results

### 3.1. Baseline Characteristics of the Participants

The research involved a total of 5,192 participants without CVD at baseline. Throughout the follow-up phase, 1382 individuals (26.6%) were found to have developed incident CVD. The participants had an average age of 58.73 ± 8.72 years at baseline, while 53.5% were female. The detailed baseline characteristics, stratified by incident CVD status and baseline obesity status, are presented in Table 1. According to recent obesity criteria, 2678 participants (51.6%) were classified as non-obese, and 2514 (48.4%) were classified as obese at baseline. The corresponding CVD incidence rates in these groups were 22.0% and 31.6%, respectively (*p* < 0.001).

### 3.2. Analysis of the Association Between Cumulative Average TyG-AIs and CVD-Related Events

**Figure 2:**
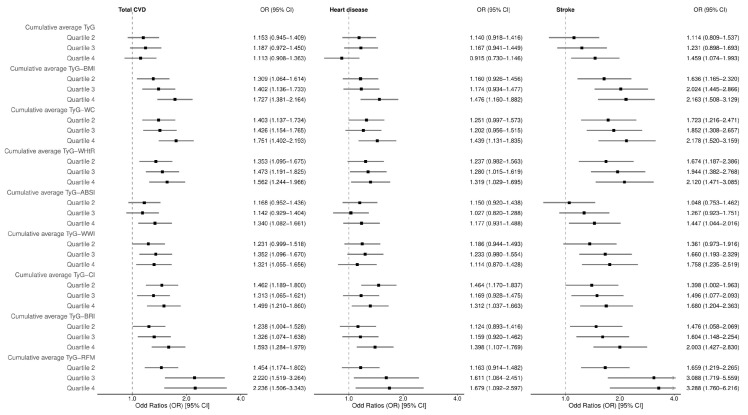
Forest plot showing the associations of cumulative average TyG-AIs with total CVD, heart disease, and stroke, analyzed using multivariable-adjusted logistic regression (Model 3, adjusted for age, sex, smoking status, alcohol consumption status, marital status, educational attainment, diabetes, and hypertension).

After full adjustment (Model 3), we examined the associations of the highest (Q4) compared to the lowest (Q1, reference) cumulative average TyG-AIs quartiles with total CVD. Cumulative average TyG alone did not demonstrate a significant association with total CVD (OR = 1.113, 95% CI: 0.908–1.363) or heart disease (OR = 0.915, 95% CI: 0.730–1.146), while a significant association was noted for stroke (OR = 1.459, 95% CI: 1.074–1.993). In contrast, all evaluated cumulative average TyG-AIs demonstrated associations of greater magnitude than TyG alone. Among these TyG-AIs, TyG-RFM exhibited the strongest associations in all CVD-related events. Specifically, for total CVD, significant associations were observed for all TyG-AIs. TyG-RFM conferred the top association strength (OR = 2.236, 95% CI: 1.506–3.343), succeeded by TyG-WC (OR = 1.751, 95% CI: 1.402–2.193). In the context of heart disease, significant associations were absent for TyG-ABSI (OR = 1.177, 95% CI: 0.931–1.488) and TyG-WWI (OR = 1.114, 95% CI: 0.870–1.428), whereas significant links were observed for the other TyG-AIs. TyG-RFM demonstrated the most substantial association (OR = 1.679, 95% CI: 1.092–2.597), while TyG-BMI exhibited a noteworthy association as well (OR = 1.476, 95% CI: 1.160–1.882). For stroke, TyG-RFM demonstrated the strongest association (OR = 3.288, 95% CI: 1.760–6.216). Other significant associations were observed for TyG-WC (OR = 2.178, 95% CI: 1.520–3.159), TyG-WWI (OR = 1.758, 95% CI: 1.235–2.519), as well as TyG-ABSI (OR = 1.447, 95% CI: 1.044–2.016). Figure 2 visually highlights these associations, demonstrating that TyG-RFM consistently yielded the highest odds ratios for all three outcomes. Detailed information is provided in Supplementary Tables S3–S5. We also evaluated the associations between the individual AIs and CVD in Model 3, and the combination of TyG and RFM still showed a stronger association with CVD (Figure S3).

**Figure 3:**
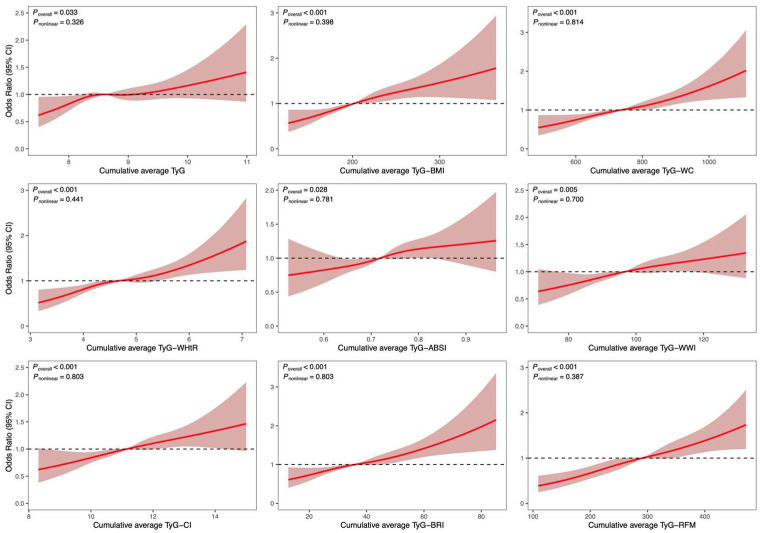
Association between cumulative average TyG-AIs with total CVD in multivariable-adjusted RCS regression Model 3. Model 3 is adjusted for age, sex, smoking status, alcohol consumption status, marital status, educational attainment, diabetes, and hypertension. The solid red lines correspond to the central estimates of odds ratios (ORs), and the red-shaded regions indicate the 95% confidence intervals. The black dashed horizontal line at OR = 1 serves as the reference level.

Fully adjusted RCS models demonstrated significant positive linear associations relating all cumulative average TyG-AIs to total CVD (*P*_overall_ < 0.05). Nonlinearity tests did not reach statistical significance (all *P*_nonlinear_ > 0.05). As shown in Figure 3, the RCS analyses visually confirmed a positive linear association between all TyG-AIs and total CVD risk, with no significant evidence of nonlinearity. No significant overall association with heart disease was noted for TyG, TyG-ABSI, TyG-WWI, and TyG-CI (*P*_overall_ > 0.05). The other composite indices maintained linear positive associations, consistent with total CVD findings. For stroke, nonlinearity was observed for TyG-BMI, TyG-WC, and TyG-WHtR (*P*_nonlinearity_ < 0.05), whereas the other indicators retained linearity (Supplementary Figures S4 and S5).

### 3.3. Receiver Operating Characteristic (ROC) Curve Analysis

The discriminative ability of each index for predicting incident CVD, heart disease, and stroke was evaluated using ROC analysis (Supplementary Figure S6). For total CVD, TyG-WC had the highest AUC (0.647), which was significantly higher than that of TyG-RFM (0.644, *p* for DeLong’s test = 0.010). For heart disease, TyG-WC and TyG-BMI shared the highest AUC (0.642), with TyG-WC’s AUC again being significantly greater than TyG-RFM’s (0.640, *p* = 0.036). Notably, for stroke, all composite indices demonstrated similar discriminative power, with no statistically significant differences observed between their AUCs when compared to TyG-RFM (all *p* > 0.05). The detailed results of DeLong’s tests are presented in Supplementary Tables S6–S8.

### 3.4. Subgroup Analysis

To explore potential heterogeneity in how cumulative average TyG-AIs are associated with CVD-related events, we conducted subgroup analyses stratified by baseline obesity status, sex, and age using a fully adjusted model (Model 3).

**Figure 4:**
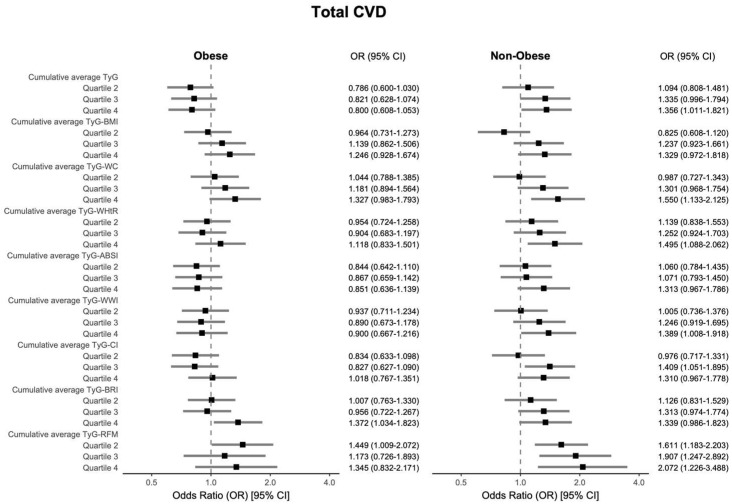
Subgroup analyses using multivariable logistic regression (Model 3) examined the associations between cumulative average TyG-AIs and total CVD in obese and non-obese subgroups, with adjustments for age, sex, smoking status, alcohol consumption status, marital status, educational attainment, diabetes, and hypertension.

**Figure 5:**
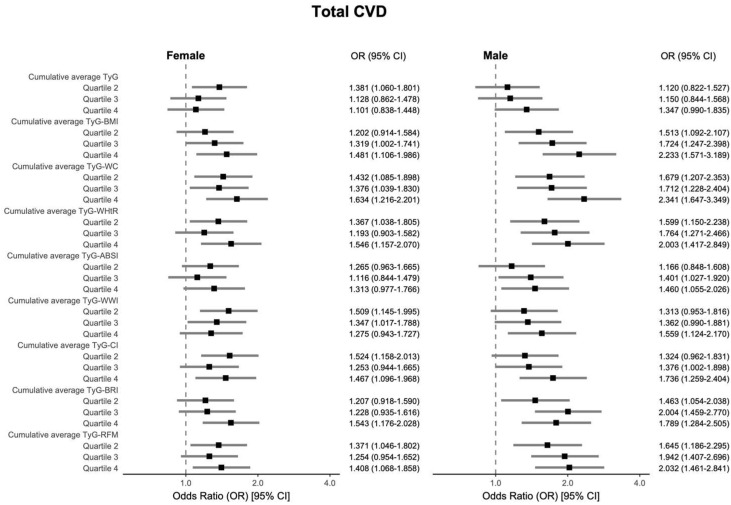
Subgroup analyses using multivariable logistic regression (Model 3) examined the associations between cumulative average TyG-AIs and total CVD in female and male subgroups, with adjustments for age, sex, smoking status, alcohol consumption status, marital status, educational attainment, diabetes, and hypertension.

**Figure 6:**
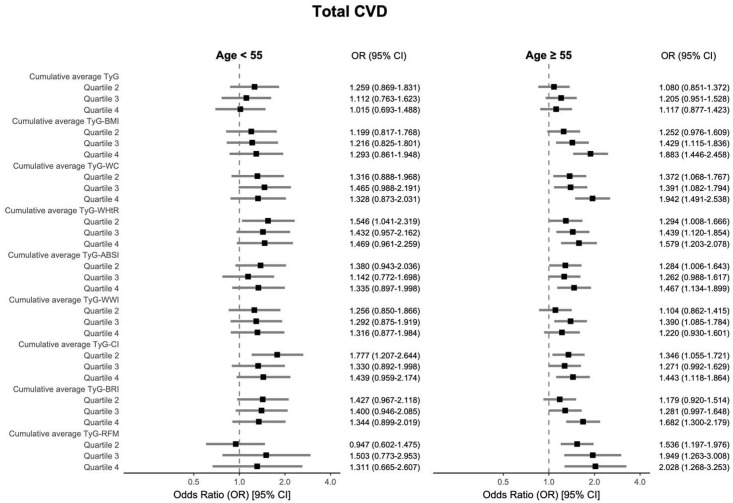
Subgroup analyses using multivariable logistic regression (Model 3) examined the associations between cumulative average TyG-AIs and total CVD in age < 55 and age ≥ 55 subgroups, with adjustments for age, sex, smoking status, alcohol consumption status, marital status, educational attainment, diabetes, and hypertension.

The results indicated that for total CVD, a significant association was observed only for TyG-BRI within the obese group (OR = 1.372, 95% CI: 1.034–1.823); among non-obese participants, TyG-RFM demonstrated the most pronounced association (OR = 2.072, 95% CI: 1.226–3.488). In sex-stratified analyses, TyG-WC showed the greatest association with total CVD in both females (OR = 1.634, 95% CI: 1.216–2.201) and males (OR = 2.341, 95% CI: 1.647–3.349). In contrast, among individuals aged ≥ 55 years, TyG-RFM showed the most significant association regarding total CVD (OR = 2.028, 95% CI: 1.268–3.253). Detailed information is shown in Figure 4, Figure 5 and Figure 6, which visually illustrate that the associations were generally stronger in non-obese participants, males, and older individuals.

For heart disease, TyG-WC was significantly associated with the outcome in the obese group (OR = 1.483, 95% CI: 1.075–2.051), whereas no significant associations were observed among non-obese participants. Within the female subgroup, TyG-WC demonstrated the highest association magnitude (OR = 1.448, 95% CI: 1.062–1.982); among male participants, TyG-RFM demonstrated the most potent association (OR = 1.627, 95% CI: 1.119–2.381). No significant associations were observed with any indices among participants aged < 55 years; in the subgroup aged ≥ 55 years, TyG-BMI exhibited the most robust association with heart disease (OR = 1.501, 95% CI: 1.130–1.998). Detailed information is shown in Supplementary Figures S7–S9.

For stroke, no significant associations were observed in the obese group. Among non-obese individuals, TyG-RFM exhibited the strongest association (OR = 3.484, 95% CI: 1.535–7.980). Among both the female subgroup and the male subgroup, the highest association magnitude was observed for TyG-WC (OR = 2.485, 95% CI: 1.500–4.237 for females; OR = 2.949, 95% CI: 1.724–5.227 for males). No significant associations were observed in the subgroup aged < 55 years. Among participants aged ≥ 55 years, the strongest association with stroke was observed for TyG-RFM (OR = 2.827, 95% CI: 1.411–5.669). Detailed information is shown in Supplementary Figures S10–S12.

## 4. Discussion

This research systematically analyzed how cumulative average TyG-AIs relate to the occurrence of incident total CVD, heart disease, and stroke. The results point to TyG-AIs exhibiting stronger associations with these CVD-related events than TyG alone. Collectively, these data imply that cumulative TyG-AIs (notably TyG-RFM) could enhance risk stratification by identifying high-risk individuals, facilitating personalized CVD prevention.

IR contributes to the pathogenesis of CVD via multiple mechanisms. It impairs vascular function, characterized by diminished nitric oxide (NO)-mediated vasodilation and augmented vascular stiffness, partly attributed to increased activity of glucocorticoid-regulated kinase 1 (SGK-1) [32]. Furthermore, IR fosters a condition of sustained, low-intensity inflammation, which includes immune cell differentiation toward a proinflammatory phenotype [33]. Additionally, IR induces metabolic dysregulation, including hyperglycemia and dyslipidemia, which subsequently promote oxidative stress, endothelial dysfunction, and atherosclerosis [5]. Obesity is acknowledged as a critical contributor to the development of IR. The underlying pathophysiology involves elevated plasma free fatty acid (FFA) levels, dysregulated adipokine secretion, and ectopic fat deposition, factors that collectively impair insulin signal transduction [34,35,36].

The TyG index, combined with AIs, recognized as effective surrogate markers for insulin resistance, has garnered increasing research interest in recent years [10,37,38]. Emerging evidence indicates that TyG-AIs are significantly associated with CVD risk. The outcomes of our current investigation concur with these previous findings. Our analyses demonstrated that cumulative average TyG-AIs, particularly TyG-RFM, exhibited stronger associations with CVD than TyG alone.

The TyG index, which serves as a surrogate marker for IR, reflects underlying pathophysiological disturbances in glucose and lipid metabolism when elevated [5]. AIs such as CI and RFM provide measures of overall adiposity and, importantly, reflect patterns of fat distribution. Notably, a robust relationship is observed between visceral adiposity and heightened metabolic risk [39,40]. Insulin resistance and adverse fat distribution patterns frequently coexist and may act synergistically, thereby contributing collectively to the progression of CVD [41,42]. Consequently, TyG-AIs function as composite metrics capable of simultaneously capturing these two critical dimensions of risk, potentially offering enhanced utility for comprehensive CVD risk stratification compared with either the TyG or AIs alone. We conducted ROC curve analysis for Model 3 using TyG-AIs as continuous variables. For incident CVD, the differences in AUCs among the indices were small (ranging from 0.639 to 0.647), with TyG-WC showing the highest AUC (0.647). We noted that the AUC of TyG-RFM was 0.644, with a difference of only 0.003 compared to TyG-WC. Although DeLong’s test indicated a statistically significant difference between the two AUCs, the actual difference in discriminative ability is negligible.

Our comparative analysis showed that the TyG-RFM composite index consistently demonstrated a stronger and more statistically robust association with all three cardiovascular outcomes than RFM alone. This finding suggests that the synergy between metabolic dysfunction (represented by TyG) and adverse body fat distribution (represented by RFM) creates a more powerful risk marker, reflecting a stronger link to the underlying pathophysiology. The observation that TyG-RFM displayed the strongest association with all three cardiovascular outcomes in our cohort is a key finding of this study, and it aligns well with the growing body of literature highlighting RFM’s clinical advantages. As detailed in the introduction, RFM was shown to have stronger associations with critical cardiometabolic conditions—including type 2 diabetes, heart failure, and NAFLD—than many traditional anthropometric indices [20,21,22]. This study significantly extends these prior findings by demonstrating that this superior associative strength persists for incident cardiovascular events (total CVD, heart disease, and stroke) in a large, prospective cohort. The superior performance of RFM may be attributed to its design, which was developed to better reflect whole-body fat percentage as measured by dual-energy X-ray absorptiometry (DEXA) [17].

Beyond statistical significance, the magnitude of these associations warrants further interpretation for their practical relevance. For total CVD, the highest quartile of TyG-RFM was associated with a more than two-fold increased risk (OR = 2.236, 95% CI: 1.506–3.343), representing a clinically meaningful, moderate-to-strong association. Notably, the effect size differed strikingly across endpoints. The OR for stroke (OR = 3.288, 95% CI: 1.760–6.216) was substantially higher than that for heart disease (OR = 1.679, 95% CI: 1.092–2.257), suggesting that the combination of insulin resistance and adiposity captured by TyG-RFM may exert a particularly potent detrimental effect on the cerebral vasculature. An association of this magnitude is of high clinical importance and provides a strong rationale for using TyG-RFM to identify high-risk individuals for targeted prevention.

Importantly, according to the fully adjusted models, TyG alone did not demonstrate a significant association with total CVD or heart disease, although a significant association with stroke persisted. This finding suggests potential heterogeneity in the strength of TyG’s association across different CVD-related events. In contrast, the combination of TyG with most AIs showed significant positive associations with CVD-related events. Exceptions occurred with TyG-ABSI and TyG-WWI, which demonstrated nonsignificant associations with heart disease specifically.

ABSI is highly independent of BMI and can serve as a tool for mortality risk stratification [43]. Combining multiple AIs that are independent of each other allows for a more comprehensive utilization of obesity-related information, such as the ARI formed by combining height, BMI, ABSI, and hip index [44]. However, the combination of such AIs with IR indicators like TyG warrants further investigation.

This study showed that the strength of the association between cumulative average TyG-AIs and CVD-related events was generally greater among non-obese individuals than among their obese counterparts. These findings suggest that TyG-AIs may possess enhanced utility for risk stratification, especially for identifying “metabolically unhealthy normal-weight” individuals, who may harbor excessive visceral or ectopic fat despite a normal BMI. This has important implications for clinical practice, as it provides a simple tool to unmask “hidden risk” in a large population subgroup often perceived as low risk, thereby enabling more targeted preventive strategies. Conversely, the attenuated association observed among obese individuals might be due to a masking effect, where the overwhelming risk contribution of obesity itself diminishes the incremental predictive value of TyG-AIs. Future research should focus on elucidating the specific determinants contributing to CVD risk within obese populations. Furthermore, the observed associations showed greater strength among males and participants aged ≥ 55 years. These subgroup differences could be attributed to factors such as sex-specific patterns of adiposity distribution, hormonal variations, and the cumulative burden of age-related CVD risk factors. However, given the exploratory nature of these subgroup analyses, these findings should be interpreted with caution and warrant confirmation in future dedicated studies.

The findings of this study provide important evidence for CVD risk stratification. Using quartile logistic regression models, we found that higher levels of TyG-RFM were strongly and significantly associated with CVD compared to lower levels of TyG-RFM, with this association being particularly pronounced in non-obese individuals and older age subgroups. These results suggest that maintaining lower TyG-RFM levels may be especially important for reducing CVD risk in non-obese and older populations.

This study has several notable strengths. Utilizing data from CHARLS, a nationally representative cohort with a prospective design, increases the statistical strength and applicability of our results to the middle-aged and older adult population of China. Employing cumulative average exposure metrics instead of single time-point measurements allowed for a more robust assessment of long-term exposure status. Furthermore, as far as we know, the present research marks the first comprehensive analysis comparing the associations of indices derived from the TyG index and eight distinct AIs in relation to the development of CVD-related events, offering fresh perspectives on the predictive capability of these indices.

However, several limitations warrant consideration. The ascertainment of CVD-related events relied primarily on participant self-reports. While this approach is common in large-scale cohort studies, it may be subject to a degree of misclassification bias. Moreover, even with the adjustment for a variety of covariates, the observational design of this research indicates that the chance of residual confounding from unmeasured or inaccurately assessed variables cannot be completely ruled out. Specifically, we did not adjust for key lifestyle factors such as dietary intake and physical activity, which could potentially confound the observed associations. Furthermore, while the clinical assessment of obesity ideally integrates multiple metrics, CHARLS offered limited indicators for assessing obesity. Our diagnosis relied solely on BMI, WC, and WHtR, which may have led to an underestimation of the true obesity prevalence. Finally, given that CHARLS consisted exclusively of Chinese adults aged 45 and older, the generalizability of our findings to younger individuals or non-Chinese populations remains to be determined and warrants further investigation.

## 5. Conclusions

Overall, this study demonstrated that TyG-AIs (especially TyG-RFM) presented stronger associations with total CVD, heart disease, and stroke than TyG alone. Lower TyG-AI levels correlate with better cardiovascular health. TyG-RFM and related indices could improve CVD risk stratification in Chinese elderly people, informing prevention and early detection strategies.

## Figures and Tables

**Table 1:** Baseline characteristics of the study participants by CVD and obesity status.

Characteristics	Non-CVD			CVD		
	Non-Obese *n* = 2090	Obese *n* = 1720	*p* Value	Non-Obese *n* = 588	Obese *n* = 794	*p* Value
Female, n (%)	876 (41.91)	1085 (63.08)	<0.001	281 (47.79)	533 (67.13)	<0.001
Age	58 (52–65)	56 (50–63)	<0.001	61 (54–67)	60 (54–65)	0.020
Education, n (%)			0.101			0.532
Primary or less	991 (47.44)	797 (46.36)		311 (52.89)	390 (49.12)	
Junior high	491 (23.50)	366 (21.29)		129 (21.94)	180 (22.67)	
Senior high	435 (20.82)	387 (22.51)		93 (15.82)	142 (17.88)	
College or more	172 (8.23)	169 (9.83)		55 (9.35)	82 (10.33)	
Married, n (%)	1777 (85.02)	1501 (87.27)	0.052	480 (81.63)	686 (86.40)	0.019
SBP, mmHg	119.50 (109.50–133.00)	128.00 (116.25–142.50)	<0.001	126.00 (113.50–141.00)	134.50 (121.00–149.50)	<0.001
DBP, mmHg	71.00 (64.00–79.00)	76.50 (69.00–84.50)	<0.001	73.00 (65.50–81.50)	78.50 (71.00–86.75)	<0.001
Ever smoked, n (%)	992 (47.56)	507 (29.48)	<0.001	269 (45.83)	228 (28.72)	<0.001
Ever drank, n (%)	914 (43.82)	584 (33.97)	<0.001	248 (42.32)	272 (34.30)	0.003
Current smoker, n (%)	835 (40.05)	387 (22.55)	<0.001	207 (35.63)	157 (19.80)	<0.001
Current drinker, n (%)	826 (39.58)	501 (29.13)	<0.001	191 (32.54)	210 (26.45)	0.016
Dyslipidemia, n (%)	59 (2.88)	171 (10.20)	<0.001	52 (9.01)	145 (18.73)	<0.001
Hypertension, n (%)	249 (12.02)	461 (26.94)	<0.001	132 (22.60)	353 (44.68)	<0.001
Diabetes, n (%)	59 (2.85)	109 (6.42)	<0.001	26 (4.45)	84 (10.71)	<0.001
Glucose, mg/dL	100.62 (92.70–109.44)	103.50 (95.76–114.84)	<0.001	101.70 (93.60–110.79)	104.94 (96.48–117.36)	<0.001
TG, mg/dL	88.50 (65.49–127.44)	121.25 (87.61–181.43)	<0.001	93.81 (69.03–138.06)	123.46 (88.50–184.08)	<0.001
TC, mg/dL	185.37 (163.15–209.54)	193.30 (169.72–219.40)	<0.001	191.56 (166.82–212.63)	196.78 (174.36–225.00)	<0.001
HDL-c, mg/dL	53.35 (44.46–63.79)	45.62 (37.50–54.51)	<0.001	53.93 (44.65–63.40)	46.01 (37.89–54.12)	<0.001
LDL-c, mg/dL	109.79 (89.30–131.06)	117.14 (95.10–140.34)	<0.001	113.66 (94.52–133.76)	121.01 (99.36–145.75)	<0.001
HbA1c, %	5.10 (4.80–5.30)	5.20 (4.90–5.50)	<0.001	5.10 (4.90–5.40)	5.20 (4.90–5.50)	<0.001
BMI, kg/m^2^	21.07 (19.60–22.38)	25.61 (24.29–27.54)	<0.001	21.26 (19.77–22.39)	26.07 (24.45–28.32)	<0.001
WC, cm	78.10 (74.00–82.20)	91.60 (88.00–96.40)	<0.001	79.00 (74.55–82.60)	93.00 (89.00–98.30)	<0.001
TyG	8.42 (8.09–8.80)	8.77 (8.40–9.22)	<0.001	8.49 (8.13–8.86)	8.79 (8.41–9.25)	<0.001
Cumulative average TyG	8.44 (8.16–8.78)	8.81 (8.48–9.21)	<0.001	8.51 (8.21–8.84)	8.85 (8.48–9.26)	<0.001
Cumulative average TyG-BMI	178.76 (165.22–194.35)	228.32 (210.68–249.62)	<0.001	182.41 (166.32–196.61)	233.34 (212.21–256.90)	<0.001
Cumulative average TyG-WC	666.74 (620.88–713.27)	812.56 (757.11–877.93)	<0.001	681.62 (630.02–723.07)	826.17 (769.45–898.88)	<0.001
Cumulative average TyG-WHtR	4.21 (3.91–4.55)	5.17 (4.78–5.59)	<0.001	4.30 (4.01–4.64)	5.27 (4.86–5.73)	<0.001
Cumulative average TyG-ABSI	0.69 (0.66–0.73)	0.74 (0.70–0.79)	<0.001	0.70 (0.67–0.75)	0.75 (0.71–0.79)	<0.001
Cumulative average TyG-WWI	91.41 (86.48–97.29)	101.71 (95.81–108.13)	<0.001	93.57 (87.85–99.36)	103.29 (96.81–109.82)	<0.001
Cumulative average TyG-CI	10.55 (10.03–11.20)	11.71 (11.08–12.41)	<0.001	10.77 (10.17–11.38)	11.86 (11.17–12.62)	<0.001
Cumulative average TyG-BRI	28.24 (23.72–33.25)	44.56 (38.80–51.84)	<0.001	29.77 (24.95–34.20)	47.08 (40.20–55.22)	<0.001
Cumulative average TyG-RFM	225.08 (186.72–306.90)	344.96 (266.51–384.29)	<0.001	241.35 (197.89–315.39)	354.44 (281.09–393.70)	<0.001

Continuous variables are presented as the mean (SD) or median (IQR) based on normality test results. Categorical variables are presented as numbers (%). SBP, systolic blood pressure; DBP, diastolic blood pressure; BMI, body mass index; WC, waist circumference; HbA1c, glycosylated hemoglobin A1c; TG, triglyceride; TC, total cholesterol; HDL-c, high-density lipoprotein cholesterol; LDL-c, low-density lipoprotein cholesterol; TyG, triglyceride–glucose index; TyG-BMI, triglyceride–glucose-body mass index; TyG-WC, triglyceride–glucose-waist circumference; TyG-WHtR, triglyceride–glucose-waist to height ratio; TyG-ABSI, triglyceride–glucose-a body shape index; TyG-WWI, triglyceride–glucose-weight-adjusted waist index; TyG-CI, triglyceride–glucose-conicity index; TyG-BRI, triglyceride–glucose-body roundness index; TyG-RFM, triglyceride–glucose-relative fat mass.

## Data Availability

The data that support the findings of this study are openly available in the CHARLS at http://charls.pku.edu.cn (accessed on 15 October 2024). Further inquiries can be directed to the corresponding author.

## Supplementary Materials

The following supporting information can be downloaded at: https://www.mdpi.com/article/10.3390/nu17132212/s1, Table S1. Correlation Matrix of Anthropometric Indicators. Table S2. Results of collinearity diagnosis in fully adjusted logistic regression models. Table S3. Association between cumulative average TyG-AIs and total CVD across multivariable-adjusted models: Model 1 (unadjusted); Model 2 (adjusted for age and sex); Model 3 (further adjusted for smoking status, alcohol consumption status, marital status, educational attainment, diabetes, and hypertension). Table S4. Association between cumulative average TyG-AIs and heart disease across multivariable-adjusted models: Model 1 (unadjusted); Model 2 (adjusted for age and sex); Model 3 (further adjusted for smoking status, alcohol consumption status, marital status, educational attainment, diabetes, and hypertension). Table S5. Association between cumulative average TyG-AIs and stroke across multivariable-adjusted models: Model 1 (unadjusted); Model 2 (adjusted for age and sex); Model 3 (further adjusted for smoking status, alcohol consumption status, marital status, educational attainment, diabetes, and hypertension). Table S6. Comparison of AUCs for Discriminating Incident Total CVD using DeLong’s Test. Table S7. Comparison of AUCs for Discriminating Incident Heart Disease using DeLong’s Test. Table S8. Comparison of AUCs for Discriminating Incident Stroke using DeLong’s Test. Figure S1. The correlations between anthropometric indicators and metabolic parameters. RFM, relative fat mass; BRI, body roundness index; CI, conicity index; WWI, weight adjusted waist index; ABSI, a body shape index; WHtR, waist to height ratio; WC, weight circumference; BMI, body mass index; TyG, triglyceride-glucose; Glu, glucose; Hba1c, glycosylated hemoglobin A1c; TC, total cholesterol; TG, triglyceride; LDL-c, low-density lipoprotein cholesterol; HDL-c, high-density lipoprotein cholesterol; Non-HDL-c, non-high-density lipoprotein cholesterol; RC, remnant cholesterol; SBP, systolic blood pressure; DBP, diastolic blood pressure; MAP, mean arterial pressure. Figure S2. Missing frequency and patterns of covariates. Figure S3. Association between cumulative average AIs with total CVD in multivariable-adjusted logistic regression Model 3. Model 3 was adjusted for age, sex, smoking status, alcohol consumption status, marital status, educational attainment, diabetes, and hypertension. Figure S4. Association between cumulative average TyG-AIs with heart disease in multivariable-adjusted RCS regression Model 3. Model 3 was adjusted for age, sex, smoking status, alcohol consumption status, marital status, educational attainment, diabetes, and hypertension. Figure S5. Association between cumulative average TyG-AIs with stroke in multivariable-adjusted RCS regression Model 3. Model 3 was adjusted for age, sex, smoking status, alcohol consumption status, marital status, educational attainment, diabetes, and hypertension. Figure S6. The ROC curves demonstrate the discriminative ability of different cumulative average TyG-AI levels for CVD-related events. Panel A shows results for total CVD, Panel B for heart disease, and Panel C for stroke. Figure S7. Subgroup analyses using multivariable logistic regression (Model 3) examined the associations between cumulative average TyG-AIs and heart disease in obese and non-obese subgroups, with adjustments for age, sex, smoking status, alcohol consumption status, marital status, educational attainment, diabetes, and hypertension. Figure S8. Subgroup analyses using multivariable logistic regression (Model 3) examined the associations between cumulative average TyG-AIs and heart disease in female and male subgroups, with adjustments for age, sex, smoking status, alcohol consumption status, marital status, educational attainment, diabetes, and hypertension. Figure S9. Subgroup analyses using multivariable logistic regression (Model 3) examined the associations between cumulative average TyG-AIs and heart disease in age < 55 and age ≥ 55 subgroups, with adjustments for age, sex, smoking status, alcohol consumption status, marital status, educational attainment, diabetes, and hypertension. Figure S10. Subgroup analyses using multivariable logistic regression (Model 3) examined the associations between cumulative average TyG-AIs and stroke in obese and non-obese subgroups, with adjustments for age, sex, smoking status, alcohol consumption status, marital status, educational attainment, diabetes, and hypertension. Figure S11. Subgroup analyses using multivariable logistic regression (Model 3) examined the associations between cumulative average TyG-AIs and stroke in female and male subgroups, with adjustments for age, sex, smoking status, alcohol consumption status, marital status, educational attainment, diabetes, and hypertension. Figure S12. Subgroup analyses using multivariable logistic regression (Model 3) examined the associations between cumulative average TyG-AIs and stroke in age < 55 and age ≥ 55 subgroups, with adjustments for age, sex, smoking status, alcohol consumption status, marital status, educational attainment, diabetes, and hypertension.

## Abbreviations

The following abbreviations are used in this manuscript:

CVD	Cardiovascular disease
IR	Insulin resistance
T2DM	Type 2 diabetes mellitus
NAFLD	Nonalcoholic fatty liver disease
TyG	Triglyceride–glucose
AIs	Anthropometric indicators
BMI	Body mass index
WC	Waist circumference
WHtR	Waist-to-height ratio
WWI	Weight-adjusted waist index
ABSI	A body shape index
BRI	Body roundness index
CI	Conicity index
RFM	Relative fat mass
TyG-AI	Triglyceride–glucose anthropometric indicator
CHARLS	China Health and Retirement Longitudinal Study
FBG	Fasting blood glucose
TG	Triglycerides
TC	Total cholesterol
SBP	Systolic blood pressure
DBP	Diastolic blood pressure
HbA1c	Glycated hemoglobin
HDL-c	High-density lipoprotein cholesterol
LDL-c	Low-density lipoprotein cholesterol
SD	Standard deviation
IQR	Interquartile range
OR	Odds ratio
95% CI	95% confidence interval
GVIF	Generalized variance inflation factor
RCS	Restricted cubic splines
ROC	Receiver operating characteristic
AUC	Area under the curve
NO	Nitric oxide
SGK-1	Serum and glucocorticoid-regulated kinase 1
FFA	Free fatty acid
