# Supplementary material for: Triglyceride–Glucose-Based Anthropometric Indices for Predicting Incident Cardiovascular Disease: Relative Fat Mass (RFM) as a Robust Indicator
Source: Nutrients. 2025 Jul 3;17(13):2212. doi: 10.3390/nu17132212 (PMC12252133; doi:10.3390/nu17132212)
Supplement: Supplementary file 1 [file nutrients-17-02212-s001.zip › Supplementary Caption.pdf]

Supplementary materials

Table S1. Correlation Matrix of Anthropometric Indicators.

Table S2. Results of collinearity diagnosis in fully adjusted logistic regression models.

Table S3. Association between cumulative average TyG-AIs and total CVD across multivariable-adjusted models: Model 1 (unadjusted); Model 2 (adjusted for age and sex); Model 3 (further adjusted for smoking status, alcohol consumption status, marital status, educational attainment, diabetes, and hypertension).

Table S4. Association between cumulative average TyG-AIs and heart disease across multivariable-adjusted models: Model 1 (unadjusted); Model 2 (adjusted for age and sex); Model 3 (further adjusted for smoking status, alcohol consumption status, marital status, educational attainment, diabetes, and hypertension).

Table S5. Association between cumulative average TyG-AIs and stroke across multivariable-adjusted models: Model 1 (unadjusted); Model 2 (adjusted for age and sex); Model 3 (further adjusted for smoking status, alcohol consumption status, marital status, educational attainment, diabetes, and hypertension).

Table S6. Comparison of AUCs for Discriminating Incident Total CVD using DeLong's Test.

Table S7. Comparison of AUCs for Discriminating Incident Heart Disease using DeLong's Test.

Table S8. Comparison of AUCs for Discriminating Incident Stroke using DeLong's Test.

Figure S1. The correlations between anthropometric indicators and metabolic parameters. RFM, relative fat mass; BRI, body roundness index; CI, conicity index; WWI, weight adjusted waist index; ABSI, a body shape index; WHtR, waist to height ratio; WC, weight circumference; BMI, body mass index; TyG, triglyceride-glucose; Glu, glucose; Hba1c, glycosylated hemoglobin A1c; TC, total cholesterol; TG, triglyceride; LDL-c, low-density lipoprotein cholesterol; HDL-c, high-density lipoprotein cholesterol; Non-HDL-c, non-high-density lipoprotein cholesterol; RC, remnant cholesterol; SBP, systolic blood

pressure; DBP, diastolic blood pressure; MAP, mean arterial pressure.

Figure S2. Missing frequency and patterns of covariates.

Figure S3. Association between cumulative average AIs with total CVD in multivariable-adjusted logistic regression Model 3. Model 3 was adjusted for age, sex, smoking status, alcohol consumption status, marital status, educational attainment, diabetes, and hypertension.

Figure S4. Association between cumulative average TyG-AIs with heart disease in multivariable-adjusted RCS regression Model 3. Model 3 was adjusted for age, sex, smoking status, alcohol consumption status, marital status, educational attainment, diabetes, and hypertension.

Figure S5. Association between cumulative average TyG-AIs with stroke in multivariable-adjusted RCS regression Model 3. Model 3 was adjusted for age, sex, smoking status, alcohol consumption status, marital status, educational attainment, diabetes, and hypertension.

Figure S6. The ROC curves demonstrate the discriminative ability of different cumulative average TyG-AI levels for CVD-related events. Panel A shows results for total CVD, Panel B for heart disease, and Panel C for stroke.

Figure S7. Subgroup analyses using multivariable logistic regression (Model 3) examined the associations between cumulative average TyG-AIs and heart disease in obese and non-obese subgroups, with adjustments for age, sex, smoking status, alcohol consumption status, marital status, educational attainment, diabetes, and hypertension.

Figure S8. Subgroup analyses using multivariable logistic regression (Model 3) examined the associations between cumulative average TyG-AIs and heart disease in female and male subgroups, with adjustments for age, sex, smoking status, alcohol consumption status, marital status, educational attainment, diabetes, and hypertension.

Figure S9. Subgroup analyses using multivariable logistic regression (Model 3) examined the associations between cumulative average TyG-AIs and heart disease in age < 55 and age ≥ 55 subgroups, with adjustments for age, sex, smoking status, alcohol consumption status, marital status, educational attainment, diabetes, and hypertension.

Figure S10. Subgroup analyses using multivariable logistic regression (Model 3) examined the associations between cumulative average TyG-AIs and stroke in obese and non-obese subgroups, with adjustments for age, sex, smoking status, alcohol consumption status, marital status, educational attainment, diabetes, and hypertension.

Figure S11. Subgroup analyses using multivariable logistic regression (Model 3) examined the associations between cumulative average TyG-AIs and stroke in female and male subgroups, with adjustments for age, sex, smoking status, alcohol consumption status, marital status, educational attainment, diabetes, and hypertension.

Figure S12. Subgroup analyses using multivariable logistic regression (Model 3) examined the associations between cumulative average TyG-AIs and stroke in age < 55 and age ≥ 55 subgroups, with adjustments for age, sex, smoking status, alcohol consumption status, marital status, educational attainment, diabetes, and hypertension.
