# Supplementary material for: Triglyceride–Glucose-Based Anthropometric Indices for Predicting Incident Cardiovascular Disease: Relative Fat Mass (RFM) as a Robust Indicator
Source: Nutrients. 2025 Jul 3;17(13):2212. doi: 10.3390/nu17132212 (PMC12252133; doi:10.3390/nu17132212)
Supplement: Supplementary file 1 [file nutrients-17-02212-s001.zip › Table S1.pdf]

	BMI	WC	WHtR	ABSI	WWI	CI	BRI	RFM
**BMI**	—	0.811	0.79	-0.039	0.294	0.326	0.79	0.541
**WC**	0.811	—	0.897	0.46	0.614	0.749	0.897	0.535
**WHtR**	0.79	0.897	—	0.493	0.795	0.774	1	0.774
**ABSI**	-0.039	0.46	0.493	—	0.853	0.915	0.493	0.349
**WWI**	0.294	0.614	0.795	0.853	—	0.927	0.795	0.696
**CI**	0.326	0.749	0.774	0.915	0.927	—	0.774	0.541
**BRI**	0.79	0.897	1	0.493	0.795	0.774	—	0.774
**RFM**	0.541	0.535	0.774	0.349	0.696	0.541	0.774	—
