# Supplementary material for: Triglyceride–Glucose-Based Anthropometric Indices for Predicting Incident Cardiovascular Disease: Relative Fat Mass (RFM) as a Robust Indicator
Source: Nutrients. 2025 Jul 3;17(13):2212. doi: 10.3390/nu17132212 (PMC12252133; doi:10.3390/nu17132212)
Supplement: Supplementary file 1 [file nutrients-17-02212-s001.zip › Table S2.pdf]

Cumulative average TyG-BMI

Variables	GVIF	Df	$GVIF^{1/2Df}$
Cumulative average TyG-BMI	1.16	3	1.02
Age	1.32	1	1.15
Gender	2.47	1	1.57
Smoke	2.05	1	1.43
Drink	1.47	1	1.21
Marriage	1.10	1	1.05
Education	1.23	1	1.11
Hypertension	1.08	1	1.04
Diabetes	1.02	1	1.01

Cumulative average TyG-WC

Variables	GVIF	Df	$GVIF^{1/2Df}$
Cumulative average TyG-WC	1.09	3	1.01
Age	1.28	1	1.13
Gender	2.47	1	1.57
Smoke	2.05	1	1.43
Drink	1.47	1	1.21
Marriage	1.10	1	1.05
Education	1.24	1	1.11
Hypertension	1.07	1	1.04
Diabetes	1.02	1	1.01

Cumulative average TyG-WHtR

Variables	GVIF	Df	$GVIF^{1/2Df}$
Cumulative average TyG-WHtR	1.15	3	1.02
Age	1.27	1	1.13
Gender	2.55	1	1.60
Smoke	2.05	1	1.43
Drink	1.47	1	1.21
Marriage	1.10	1	1.05
Education	1.23	1	1.11
Hypertension	1.07	1	1.03
Diabetes	1.02	1	1.01

Cumulative average TyG-ABSI

Variables	GVIF	Df	$GVIF^{1/2Df}$
Cumulative average TyG-ABSI	1.09	3	1.01
Age	1.31	1	1.14
Gender	2.52	1	1.59
Smoke	2.05	1	1.43
Drink	1.47	1	1.21
Marriage	1.10	1	1.05
Education	1.23	1	1.11
Hypertension	1.03	1	1.02
Diabetes	1.03	1	1.02

Cumulative average TyG-WWI

Variables	GVIF	Df	$GVIF^{1/2Df}$
Cumulative average TyG-WWI	1.18	3	1.03
Age	1.31	1	1.14
Gender	2.62	1	1.62
Smoke	2.05	1	1.43
Drink	1.47	1	1.21
Marriage	1.10	1	1.05
Education	1.23	1	1.11
Hypertension	1.04	1	1.02
Diabetes	1.03	1	1.01

Cumulative average TyG-CI

Variables	GVIF	Df	$GVIF^{1/2Df}$
Cumulative average TyG-CI	1.09	3	1.01
Age	1.28	1	1.13
Gender	2.52	1	1.59
Smoke	2.05	1	1.43
Drink	1.47	1	1.21
Marriage	1.10	1	1.05
Education	1.23	1	1.11
Hypertension	1.05	1	1.02
Diabetes	1.03	1	1.02

Cumulative average TyG-BRI			
Variables	GVIF	Df	$GVIF^{1/2Df}$
Cumulative average TyG-BRI	1.17	3	1.03
Age	1.27	1	1.13
Gender	2.56	1	1.60
Smoke	2.06	1	1.43
Drink	1.47	1	1.21
Marriage	1.10	1	1.05
Education	1.24	1	1.11
Hypertension	1.07	1	1.03
Diabetes	1.02	1	1.01

Cumulative average TyG-RFM

Variables	GVIF	Df	$GVIF^{1/2Df}$
Cumulative average TyG-RFM	6.11	3	1.35
Age	1.27	1	1.13
Gender	7.22	1	2.69
Smoke	2.06	1	1.44
Drink	1.47	1	1.21
Marriage	1.10	1	1.05
Education	1.23	1	1.11
Hypertension	1.06	1	1.03
Diabetes	1.02	1	1.01

Cumulative average TyG

Variables	GVIF	Df	$GVIF^{1/2Df}$
Cumulative average TyG	1.05	3	1.01
Age	1.27	1	1.13
Gender	2.49	1	1.58
Smoke	2.05	1	1.43
Drink	1.47	1	1.21
Marriage	1.10	1	1.05
Education	1.23	1	1.11
Hypertension	1.04	1	1.02
Diabetes	1.04	1	1.02
