# Supplementary material for: Triglyceride–Glucose-Based Anthropometric Indices for Predicting Incident Cardiovascular Disease: Relative Fat Mass (RFM) as a Robust Indicator
Source: Nutrients. 2025 Jul 3;17(13):2212. doi: 10.3390/nu17132212 (PMC12252133; doi:10.3390/nu17132212)
Supplement: Supplementary file 1 [file nutrients-17-02212-s001.zip › Table S3.pdf]

Variables	Quartile	Model 1		Model 2		Model 3	
		OR (95% CI)	*P*-value	OR (95% CI)	*P*-value	OR (95% CI)	*P*-value
Cumulative average TyG	Q2	1.22 (1.00-1.48)	0.048	1.21 (1.00-1.47)	0.056	1.15 (0.94-1.41)	0.162
	Q3	1.28 (1.05-1.55)	0.014	1.28 (1.05-1.56)	0.013	1.19 (0.97-1.45)	0.092
	Q4	1.25 (1.03-1.52)	0.023	1.27 (1.05-1.55)	0.016	1.11 (0.91-1.36)	0.303
Cumulative average TyG-BMI	Q2	1.30 (1.07-1.60)	0.010	1.38 (1.13-1.70)	0.002	1.31 (1.06-1.61)	0.011
	Q3	1.43 (1.17-1.75)	<0.001	1.61 (1.31-1.97)	<0.001	1.40 (1.14-1.73)	0.002
	Q4	1.81 (1.48-2.23)	<0.001	2.13 (1.72-2.65)	<0.001	1.73 (1.38-2.16)	<0.001
Cumulative average TyG-WC	Q2	1.46 (1.19-1.80)	<0.001	1.48 (1.20-1.82)	<0.001	1.40 (1.14-1.73)	0.002
	Q3	1.59 (1.29-1.95)	<0.001	1.64 (1.33-2.02)	<0.001	1.43 (1.15-1.77)	0.001
	Q4	2.02 (1.63-2.50)	<0.001	2.15 (1.74-2.67)	<0.001	1.75 (1.40-2.19)	<0.001
Cumulative average TyG-WHtR	Q2	1.46 (1.19-1.80)	<0.001	1.45 (1.18-1.79)	<0.001	1.35 (1.10-1.67)	0.005
	Q3	1.70 (1.38-2.08)	<0.001	1.66 (1.35-2.04)	<0.001	1.47 (1.19-1.83)	<0.001
	Q4	1.99 (1.62-2.46)	<0.001	1.92 (1.54-2.40)	<0.001	1.56 (1.24-1.97)	<0.001
Cumulative average TyG-ABSI	Q2	1.30 (1.07-1.59)	0.010	1.22 (1.00-1.50)	0.052	1.17 (0.95-1.44)	0.139
	Q3	1.34 (1.10-1.64)	0.004	1.23 (1.00-1.51)	0.046	1.14 (0.93-1.40)	0.208
	Q4	1.76 (1.44-2.15)	<0.001	1.51 (1.23-1.86)	<0.001	1.34 (1.08-1.66)	0.007
Cumulative average TyG-WWI	Q2	1.37 (1.12-1.68)	0.002	1.31 (1.07-1.61)	0.011	1.23 (1.00-1.52)	0.052
	Q3	1.68 (1.37-2.05)	<0.001	1.52 (1.24-1.87)	<0.001	1.35 (1.10-1.67)	0.005
	Q4	1.78 (1.45-2.19)	<0.001	1.52 (1.22-1.90)	<0.001	1.32 (1.05-1.66)	0.016
Cumulative average TyG-CI	Q2	1.60 (1.31-1.96)	<0.001	1.56 (1.28-1.92)	<0.001	1.46 (1.19-1.80)	<0.001
	Q3	1.63 (1.33-1.99)	<0.001	1.50 (1.22-1.84)	<0.001	1.31 (1.06-1.62)	0.011
	Q4	1.96 (1.60-2.41)	<0.001	1.77 (1.44-2.19)	<0.001	1.50 (1.21-1.86)	<0.001
Cumulative average TyG-BRI	Q2	1.35 (1.10-1.66)	0.004	1.31 (1.07-1.61)	0.010	1.24 (1.00-1.53)	0.046
	Q3	1.53 (1.26-1.88)	<0.001	1.50 (1.22-1.84)	<0.001	1.33 (1.07-1.64)	0.009
	Q4	2.03 (1.67-2.48)	<0.001	1.92 (1.56-2.37)	<0.001	1.59 (1.28-1.98)	<0.001
Cumulative average TyG-RFM	Q2	1.43 (1.17-1.75)	<0.001	1.63 (1.32-2.01)	<0.001	1.45 (1.17-1.80)	<0.001
	Q3	1.54 (1.27-1.88)	<0.001	2.73 (1.89-3.97)	<0.001	2.22 (1.52-3.26)	<0.001
	Q4	1.74 (1.43-2.12)	<0.001	3.06 (2.10-4.52)	<0.001	2.24 (1.51-3.34)	<0.001

A multivariate logistic regression model was used to assess the associations between cumulative average TyG-AIs (grouped by quartiles) and total CVD. Using the lowest quartile (Q1) as the reference group, the odds ratios (ORs), 95% confidence intervals (CIs), and P-values for the remaining quartiles (Q2, Q3, Q4) were calculated and reported. Three progressively adjusted models were constructed: Model 1 was unadjusted; Model 2 adjusted for age and sex; Model 3 further adjusted for smoking status, alcohol consumption status, marital status, educational attainment, diabetes, and hypertension based on Model 2.
