# Supplementary material for: Triglyceride–Glucose-Based Anthropometric Indices for Predicting Incident Cardiovascular Disease: Relative Fat Mass (RFM) as a Robust Indicator
Source: Nutrients. 2025 Jul 3;17(13):2212. doi: 10.3390/nu17132212 (PMC12252133; doi:10.3390/nu17132212)
Supplement: Supplementary file 1 [file nutrients-17-02212-s001.zip › Table S4.pdf]

Variables	Quartile	Model 1		Model 2		Model 3	
		OR (95% CI)	*P*-value	OR (95% CI)	*P*-value	OR (95% CI)	*P*-value
Cumulative average TyG	Q2	1.21 (0.98-1.49)	0.077	1.20 (0.97-1.48)	0.098	1.14 (0.92-1.42)	0.235
	Q3	1.26 (1.02-1.55)	0.033	1.24 (1.01-1.54)	0.045	1.17 (0.94-1.45)	0.160
	Q4	1.06 (0.85-1.31)	0.622	1.06 (0.85-1.31)	0.626	0.92 (0.73-1.15)	0.440
Cumulative average TyG-BMI	Q2	1.18 (0.95-1.47)	0.148	1.23 (0.98-1.53)	0.074	1.16 (0.93-1.46)	0.199
	Q3	1.25 (1.01-1.56)	0.043	1.35 (1.08-1.69)	0.008	1.17 (0.93-1.48)	0.171
	Q4	1.62 (1.30-2.02)	<0.001	1.81 (1.44-2.28)	<0.001	1.48 (1.16-1.88)	0.002
Cumulative average TyG-WC	Q2	1.32 (1.05-1.65)	0.016	1.32 (1.05-1.65)	0.016	1.25 (1.00-1.57)	0.054
	Q3	1.38 (1.10-1.72)	0.005	1.39 (1.11-1.74)	0.004	1.20 (0.96-1.52)	0.117
	Q4	1.68 (1.34-2.11)	<0.001	1.76 (1.40-2.23)	<0.001	1.44 (1.13-1.83)	0.003
Cumulative average TyG-WHtR	Q2	1.41 (1.13-1.78)	0.003	1.34 (1.06-1.68)	0.013	1.24 (0.98-1.56)	0.073
	Q3	1.58 (1.27-1.98)	<0.001	1.45 (1.15-1.82)	0.002	1.28 (1.02-1.62)	0.038
	Q4	1.85 (1.47-2.33)	<0.001	1.62 (1.28-2.06)	<0.001	1.32 (1.03-1.69)	0.029
Cumulative average TyG-ABSI	Q2	1.30 (1.05-1.62)	0.018	1.21 (0.97-1.51)	0.092	1.15 (0.92-1.44)	0.221
	Q3	1.22 (0.98-1.53)	0.071	1.10 (0.88-1.37)	0.418	1.03 (0.82-1.29)	0.819
	Q4	1.58 (1.26-1.97)	<0.001	1.32 (1.05-1.66)	0.017	1.18 (0.93-1.49)	0.173
Cumulative average TyG-WWI	Q2	1.36 (1.08-1.70)	0.008	1.25 (1.00-1.57)	0.055	1.19 (0.94-1.49)	0.144
	Q3	1.63 (1.31-2.03)	<0.001	1.38 (1.10-1.73)	0.005	1.23 (0.98-1.55)	0.075
	Q4	1.64 (1.31-2.06)	<0.001	1.28 (1.01-1.63)	0.044	1.11 (0.87-1.43)	0.393
Cumulative average TyG-CI	Q2	1.63 (1.31-2.04)	<0.001	1.57 (1.25-1.96)	<0.001	1.46 (1.17-1.84)	<0.001
	Q3	1.50 (1.20-1.88)	<0.001	1.34 (1.07-1.68)	0.012	1.17 (0.93-1.48)	0.185
	Q4	1.77 (1.41-2.22)	<0.001	1.55 (1.23-1.95)	<0.001	1.31 (1.04-1.66)	0.024
Cumulative average TyG-BRI	Q2	1.28 (1.03-1.61)	0.028	1.20 (0.96-1.51)	0.115	1.12 (0.89-1.42)	0.321
	Q3	1.43 (1.15-1.79)	0.001	1.32 (1.05-1.65)	0.017	1.16 (0.92-1.46)	0.211
	Q4	1.94 (1.56-2.41)	<0.001	1.68 (1.34-2.11)	<0.001	1.40 (1.11-1.77)	0.005
Cumulative average TyG-RFM	Q2	1.24 (0.99-1.56)	0.062	1.32 (1.04-1.67)	0.021	1.16 (0.91-1.48)	0.220
	Q3	1.62 (1.30-2.02)	<0.001	2.03 (1.36-3.05)	<0.001	1.61 (1.06-2.45)	0.025
	Q4	1.89 (1.52-2.34)	<0.001	2.32 (1.53-3.53)	<0.001	1.68 (1.09-2.60)	0.019

A multivariate logistic regression model was used to assess the associations between cumulative average TyG-AIs (grouped by quartiles) and heart disease. Using the lowest quartile (Q1) as the reference group, the odds ratios (ORs), 95% confidence intervals (CIs), and P-values for the remaining quartiles (Q2, Q3, Q4) were calculated and reported. Three progressively adjusted models were constructed: Model 1 was unadjusted; Model 2 adjusted for age and sex; Model 3 further adjusted for smoking status, alcohol consumption status, marital status, educational attainment, diabetes, and hypertension based on Model 2.
