# Supplementary material for: Triglyceride–Glucose-Based Anthropometric Indices for Predicting Incident Cardiovascular Disease: Relative Fat Mass (RFM) as a Robust Indicator
Source: Nutrients. 2025 Jul 3;17(13):2212. doi: 10.3390/nu17132212 (PMC12252133; doi:10.3390/nu17132212)
Supplement: Supplementary file 1 [file nutrients-17-02212-s001.zip › Table S5.pdf]

Variables	Quartile	Model 1		Model 2		Model 3	
		OR (95% CI)	*P*-value	OR (95% CI)	*P*-value	OR (95% CI)	*P*-value
Cumulative average TyG	Q2	1.17 (0.85-1.60)	0.335	1.17 (0.86-1.62)	0.319	1.11 (0.81-1.54)	0.508
	Q3	1.30 (0.95-1.77)	0.100	1.34 (0.99-1.84)	0.063	1.23 (0.90-1.69)	0.198
	Q4	1.59 (1.18-2.15)	0.002	1.65 (1.22-2.24)	0.001	1.46 (1.07-1.99)	0.016
Cumulative average TyG-BMI	Q2	1.58 (1.13-2.24)	0.008	1.71 (1.22-2.42)	0.002	1.64 (1.16-2.32)	0.005
	Q3	1.91 (1.38-2.67)	<0.001	2.25 (1.62-3.17)	<0.001	2.02 (1.44-2.87)	<0.001
	Q4	2.07 (1.48-2.92)	<0.001	2.65 (1.87-3.78)	<0.001	2.16 (1.51-3.13)	<0.001
Cumulative average TyG-WC	Q2	1.73 (1.23-2.47)	0.002	1.80 (1.27-2.57)	0.001	1.72 (1.22-2.47)	0.003
	Q3	1.91 (1.36-2.71)	<0.001	2.05 (1.45-2.92)	<0.001	1.85 (1.31-2.66)	<0.001
	Q4	2.47 (1.75-3.52)	<0.001	2.67 (1.89-3.82)	<0.001	2.18 (1.52-3.16)	<0.001
Cumulative average TyG-WHtR	Q2	1.58 (1.13-2.23)	0.009	1.74 (1.24-2.47)	0.002	1.67 (1.19-2.39)	0.004
	Q3	1.89 (1.36-2.65)	<0.001	2.10 (1.51-2.98)	<0.001	1.94 (1.38-2.77)	<0.001
	Q4	2.13 (1.52-3.01)	<0.001	2.57 (1.81-3.68)	<0.001	2.12 (1.47-3.08)	<0.001
Cumulative average TyG-ABSI	Q2	1.09 (0.79-1.52)	0.593	1.07 (0.77-1.48)	0.705	1.05 (0.75-1.46)	0.783
	Q3	1.43 (1.05-1.96)	0.025	1.38 (1.01-1.90)	0.047	1.27 (0.92-1.75)	0.147
	Q4	1.78 (1.31-2.44)	<0.001	1.66 (1.21-2.30)	0.002	1.45 (1.04-2.02)	0.028
Cumulative average TyG-WWI	Q2	1.40 (1.01-1.96)	0.048	1.46 (1.04-2.04)	0.028	1.36 (0.97-1.92)	0.074
	Q3	1.75 (1.27-2.42)	<0.001	1.85 (1.33-2.58)	<0.001	1.66 (1.19-2.33)	0.003
	Q4	1.89 (1.37-2.63)	<0.001	2.06 (1.46-2.92)	<0.001	1.76 (1.24-2.52)	0.002
Cumulative average TyG-CI	Q2	1.43 (1.03-2.00)	0.034	1.46 (1.05-2.05)	0.025	1.40 (1.00-1.96)	0.050
	Q3	1.67 (1.21-2.32)	0.002	1.67 (1.21-2.32)	0.002	1.50 (1.08-2.09)	0.017
	Q4	2.01 (1.46-2.78)	<0.001	2.00 (1.45-2.79)	<0.001	1.68 (1.20-2.36)	0.003
Cumulative average TyG-BRI	Q2	1.44 (1.04-2.01)	0.028	1.51 (1.09-2.11)	0.014	1.48 (1.06-2.07)	0.023
	Q3	1.56 (1.13-2.16)	0.007	1.75 (1.26-2.43)	<0.001	1.60 (1.15-2.25)	0.006
	Q4	2.03 (1.49-2.79)	<0.001	2.41 (1.74-3.36)	<0.001	2.00 (1.43-2.83)	<0.001
Cumulative average TyG-RFM	Q2	1.53 (1.14-2.07)	0.005	1.82 (1.35-2.47)	<0.001	1.66 (1.22-2.26)	0.001
	Q3	1.08 (0.79-1.48)	0.639	3.52 (2.00-6.22)	<0.001	3.09 (1.72-5.56)	<0.001
	Q4	1.24 (0.92-1.69)	0.164	4.29 (2.35-7.92)	<0.001	3.29 (1.76-6.22)	<0.001

A multivariate logistic regression model was used to assess the associations between cumulative average TyG-AIs (grouped by quartiles) and stroke. Using the lowest quartile (Q1) as the reference group, the odds ratios (ORs), 95% confidence intervals (CIs), and P-values for the remaining quartiles (Q2, Q3, Q4) were calculated and reported. Three progressively adjusted models were constructed: Model 1 was unadjusted; Model 2 adjusted for age and sex; Model 3 further adjusted for smoking status, alcohol consumption status, marital status, educational attainment, diabetes, and hypertension based on Model 2.
