# Supplementary material for: Triglyceride–Glucose-Based Anthropometric Indices for Predicting Incident Cardiovascular Disease: Relative Fat Mass (RFM) as a Robust Indicator
Source: Nutrients. 2025 Jul 3;17(13):2212. doi: 10.3390/nu17132212 (PMC12252133; doi:10.3390/nu17132212)
Supplement: Supplementary file 1 [file nutrients-17-02212-s001.zip › Table S6.pdf]

Indicator	AUC (95% CI)	*P*-value (vs TyG-RFM)
Cumulative average TyG-WC	0.647 (0.630 - 0.664)	0.010
Cumulative average TyG-BMI	0.646 (0.629 - 0.663)	0.198
Cumulative average TyG-BRI	0.645 (0.628 - 0.662)	0.098
Cumulative average TyG-WHtR	0.645 (0.628 - 0.662)	0.125
Cumulative average TyG-RFM	0.644 (0.627 - 0.661)	Reference
Cumulative average TyG-CI	0.641 (0.624 - 0.658)	0.125
Cumulative average TyG-WWI	0.640 (0.623 - 0.657)	0.029
Cumulative average TyG-ABSI	0.639 (0.622 - 0.656)	0.042
Cumulative average TyG	0.639 (0.622 - 0.656)	0.059
