# Supplementary material for: Triglyceride–Glucose-Based Anthropometric Indices for Predicting Incident Cardiovascular Disease: Relative Fat Mass (RFM) as a Robust Indicator
Source: Nutrients. 2025 Jul 3;17(13):2212. doi: 10.3390/nu17132212 (PMC12252133; doi:10.3390/nu17132212)
Supplement: Supplementary file 1 [file nutrients-17-02212-s001.zip › Table S7.pdf]

Indicator	AUC (95% CI)	*P*-value (vs TyG-RFM)
Cumulative average TyG-WC	0.642 (0.624 - 0.660)	0.036
Cumulative average TyG-BMI	0.642 (0.623 - 0.660)	0.109
Cumulative average TyG-BRI	0.641 (0.623 - 0.660)	0.072
Cumulative average TyG-WHtR	0.640 (0.622 - 0.659)	0.211
Cumulative average TyG-RFM	0.640 (0.621 - 0.658)	Reference
Cumulative average TyG-CI	0.638 (0.619 - 0.656)	0.137
Cumulative average TyG-WWI	0.637 (0.619 - 0.656)	0.099
Cumulative average TyG-ABSI	0.637 (0.618 - 0.655)	0.124
Cumulative average TyG	0.637 (0.618 - 0.655)	0.142
