# Supplementary material for: Triglyceride–Glucose-Based Anthropometric Indices for Predicting Incident Cardiovascular Disease: Relative Fat Mass (RFM) as a Robust Indicator
Source: Nutrients. 2025 Jul 3;17(13):2212. doi: 10.3390/nu17132212 (PMC12252133; doi:10.3390/nu17132212)
Supplement: Supplementary file 1 [file nutrients-17-02212-s001.zip › Table S8.pdf]

Indicator	AUC (95% CI)	*P*-value (vs TyG-RFM)
Cumulative average TyG-WHtR	0.661 (0.636 - 0.686)	0.467
Cumulative average TyG-WC	0.661 (0.636 - 0.685)	0.839
Cumulative average TyG-RFM	0.660 (0.636 - 0.685)	Reference
Cumulative average TyG-BRI	0.659 (0.634 - 0.684)	0.540
Cumulative average TyG-CI	0.658 (0.633 - 0.683)	0.541
Cumulative average TyG-WWI	0.658 (0.633 - 0.683)	0.490
Cumulative average TyG-BMI	0.657 (0.632 - 0.682)	0.380
Cumulative average TyG-ABSI	0.654 (0.629 - 0.679)	0.191
Cumulative average TyG	0.654 (0.628 - 0.679)	0.234
