# Supplementary figures and images for: Triglyceride–Glucose-Based Anthropometric Indices for Predicting Incident Cardiovascular Disease: Relative Fat Mass (RFM) as a Robust Indicator
Source: Nutrients. 2025 Jul 3;17(13):2212. doi: 10.3390/nu17132212 (PMC12252133; doi:10.3390/nu17132212)

# Stroke

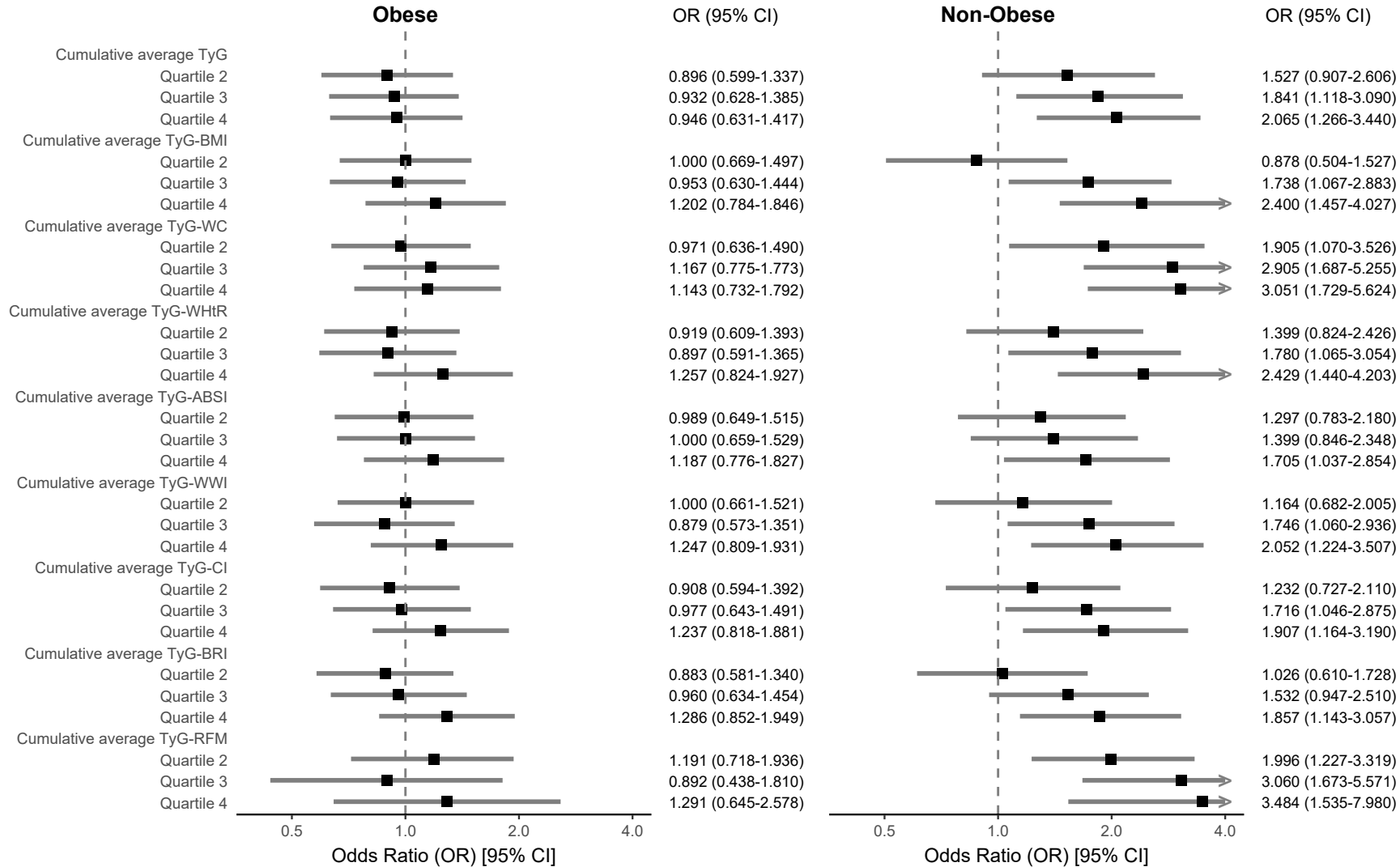

Supplement: Supplementary file 1 [file nutrients-17-02212-s001.zip › Figure S10.pdf]

# Stroke

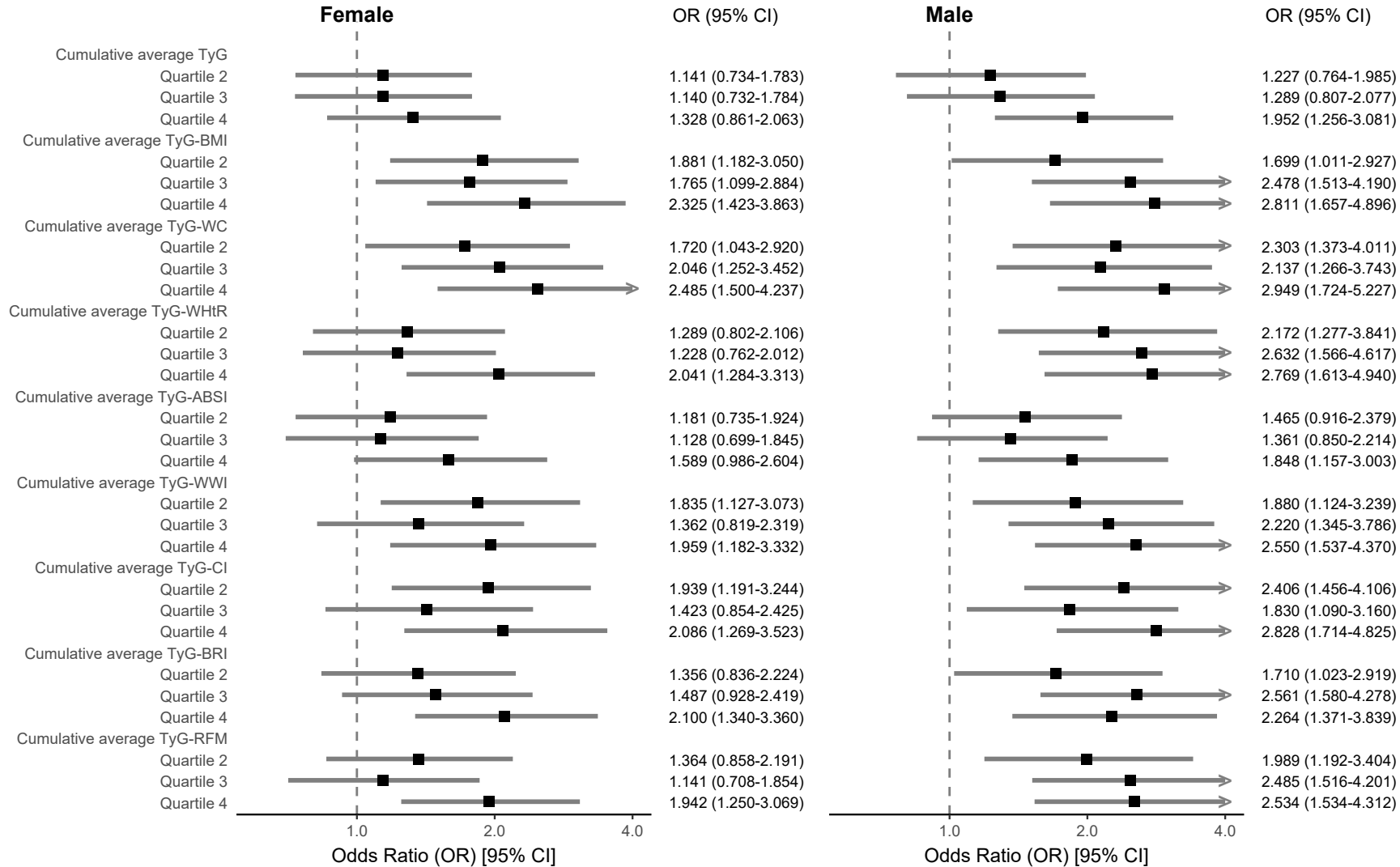

Supplement: Supplementary file 1 [file nutrients-17-02212-s001.zip › Figure S11.pdf]

# Stroke

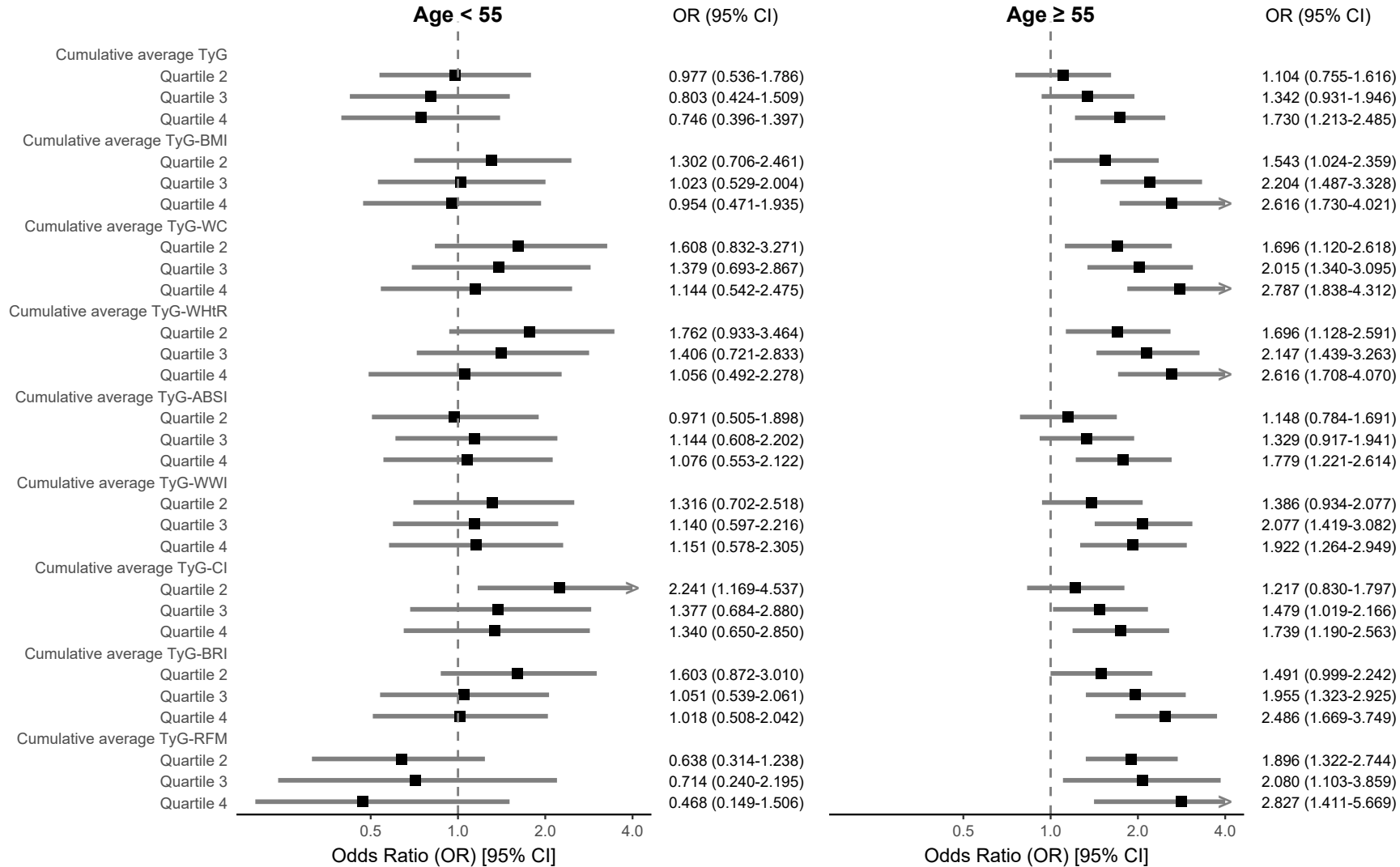

Supplement: Supplementary file 1 [file nutrients-17-02212-s001.zip › Figure S12.pdf]

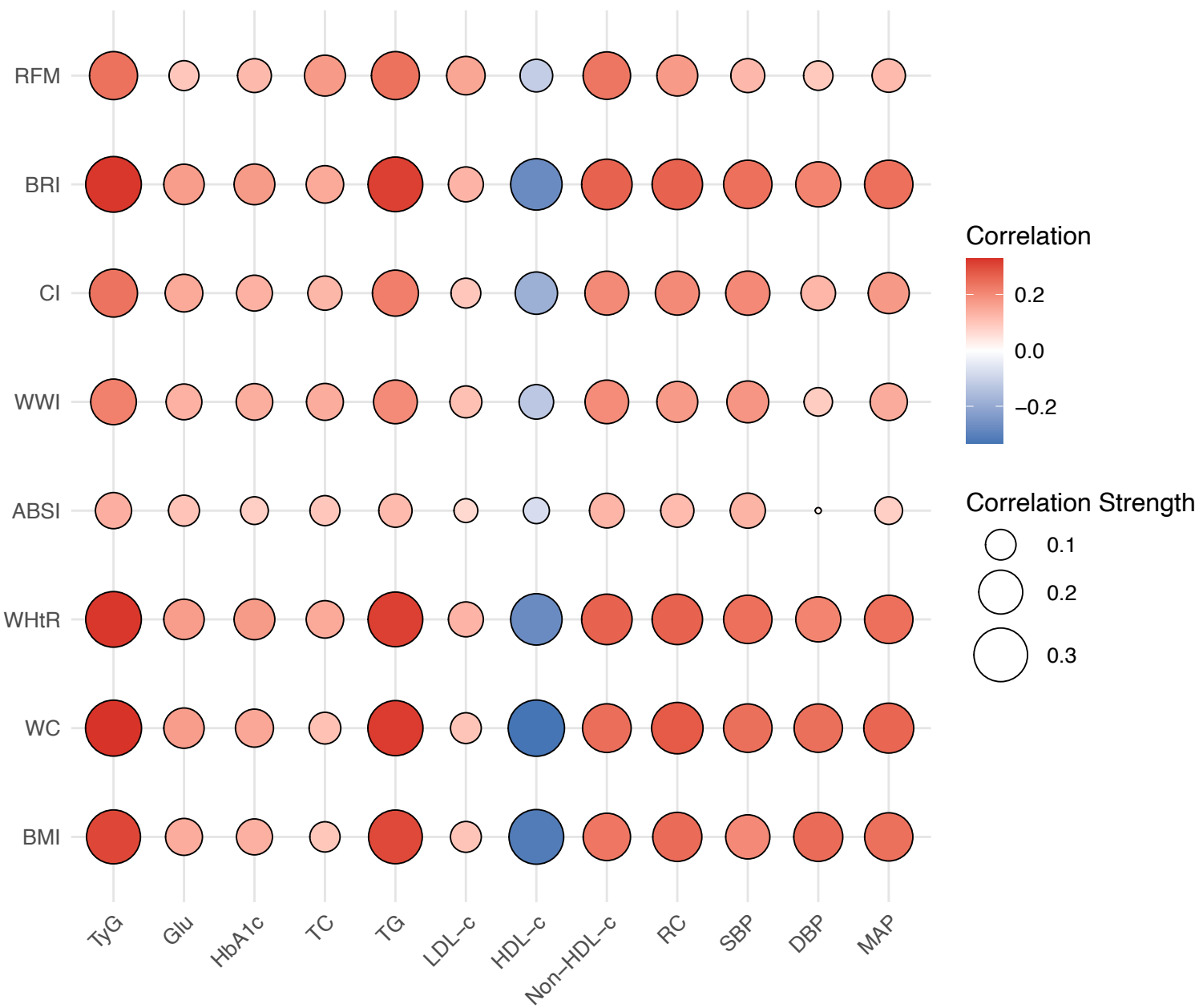

Supplement: Supplementary file 1 [file nutrients-17-02212-s001.zip › Figure S1.pdf]

Percentage of Missing Values

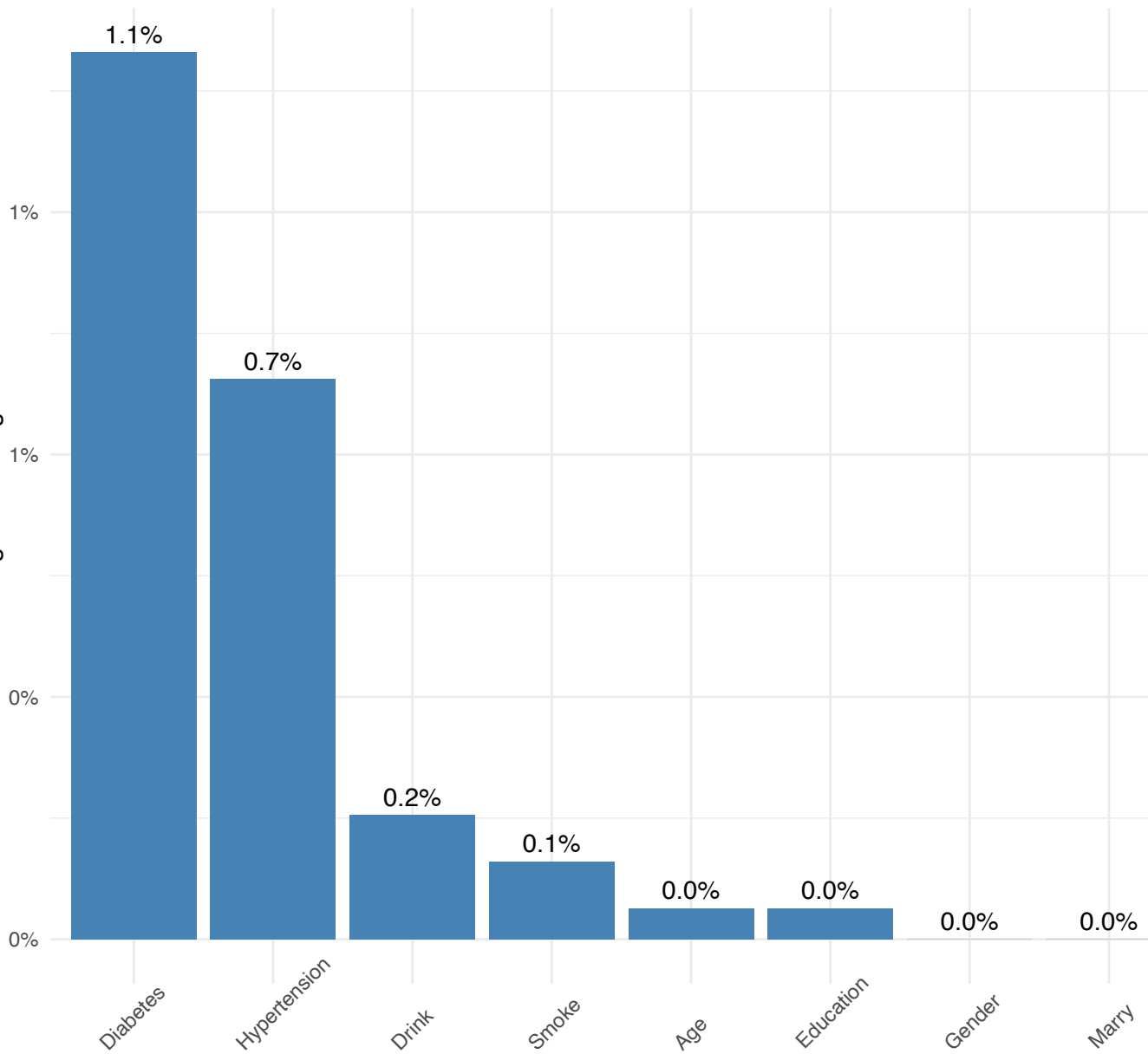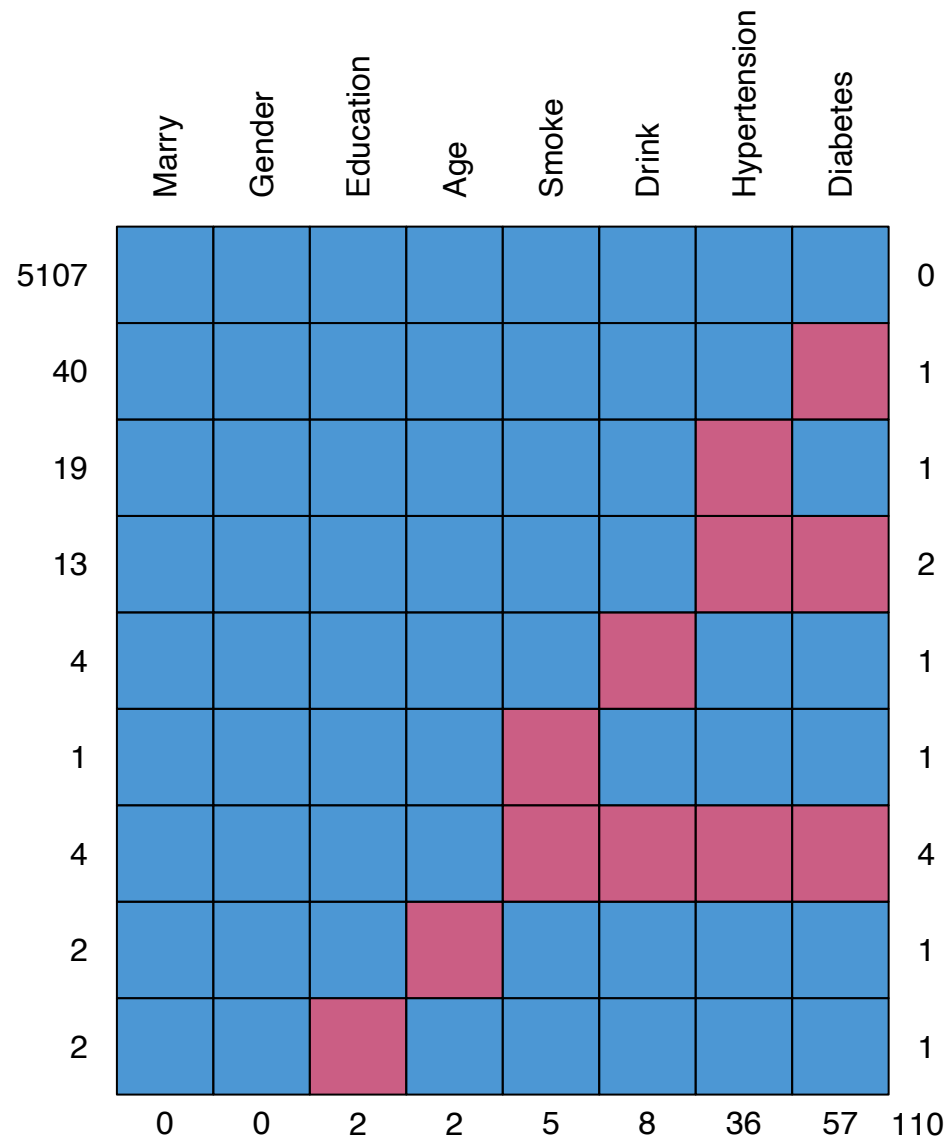

Supplement: Supplementary file 1 [file nutrients-17-02212-s001.zip › Figure S2.pdf]

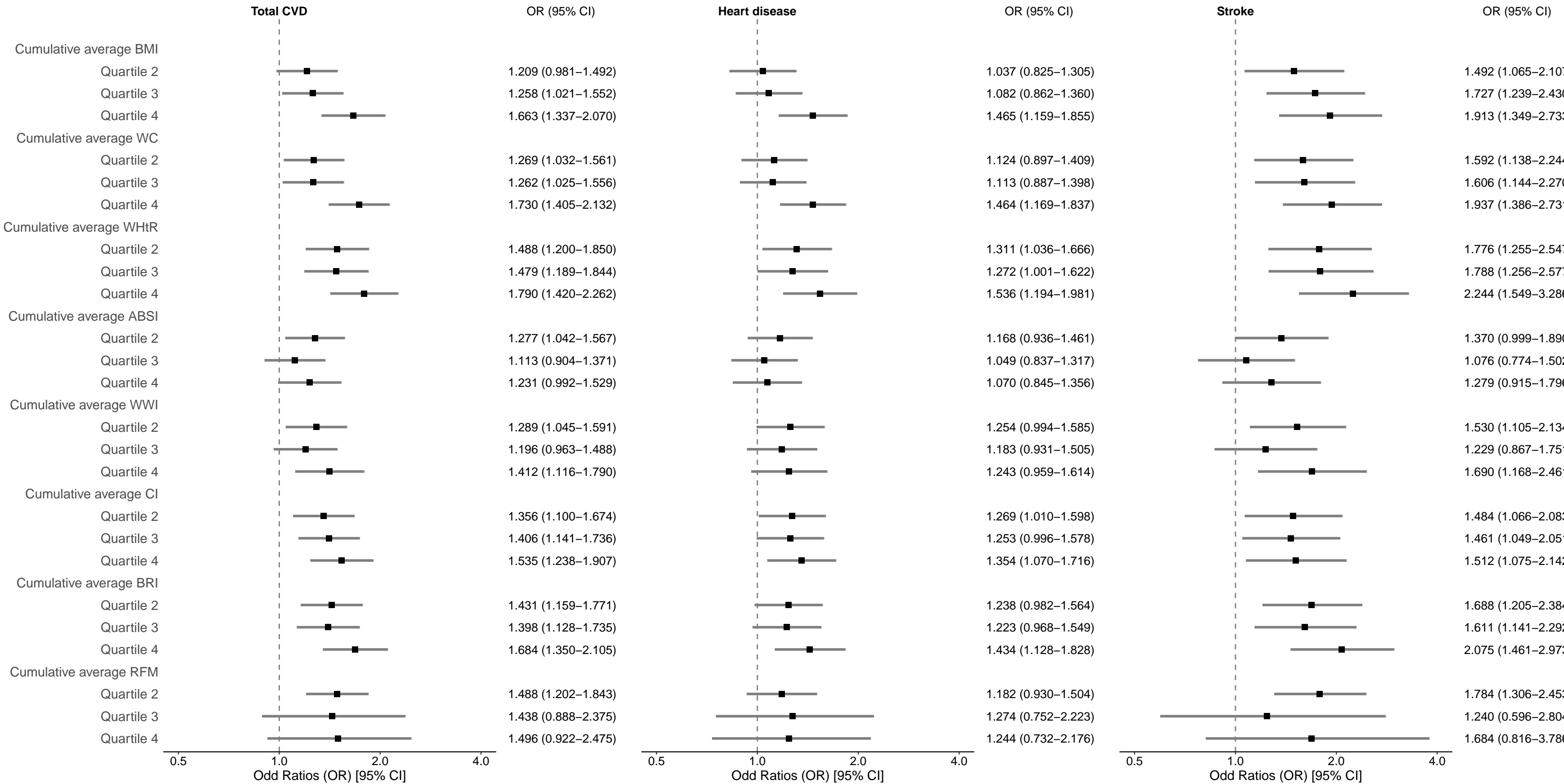

Supplement: Supplementary file 1 [file nutrients-17-02212-s001.zip › Figure S3.pdf]

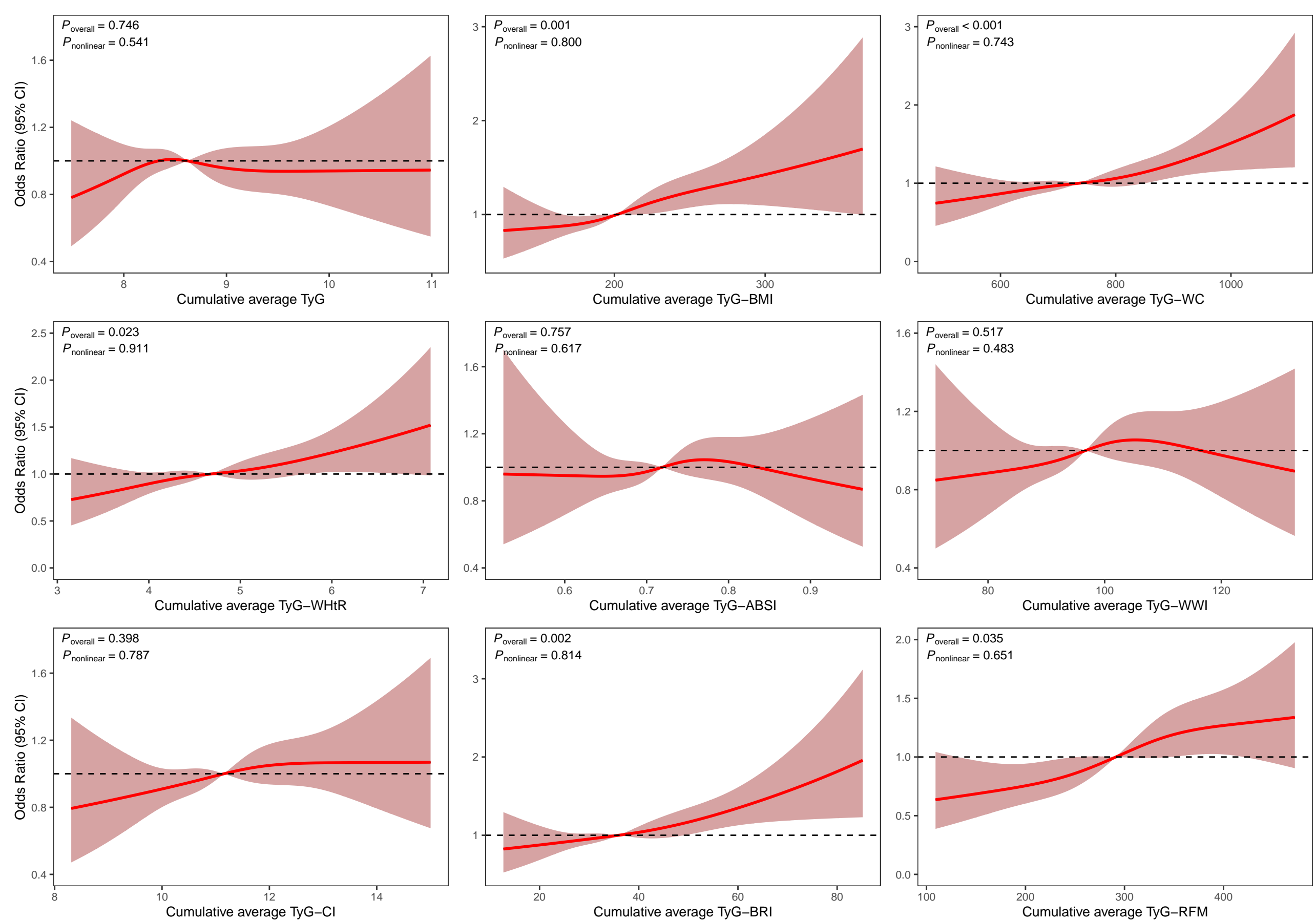

Supplement: Supplementary file 1 [file nutrients-17-02212-s001.zip › Figure S4.pdf]

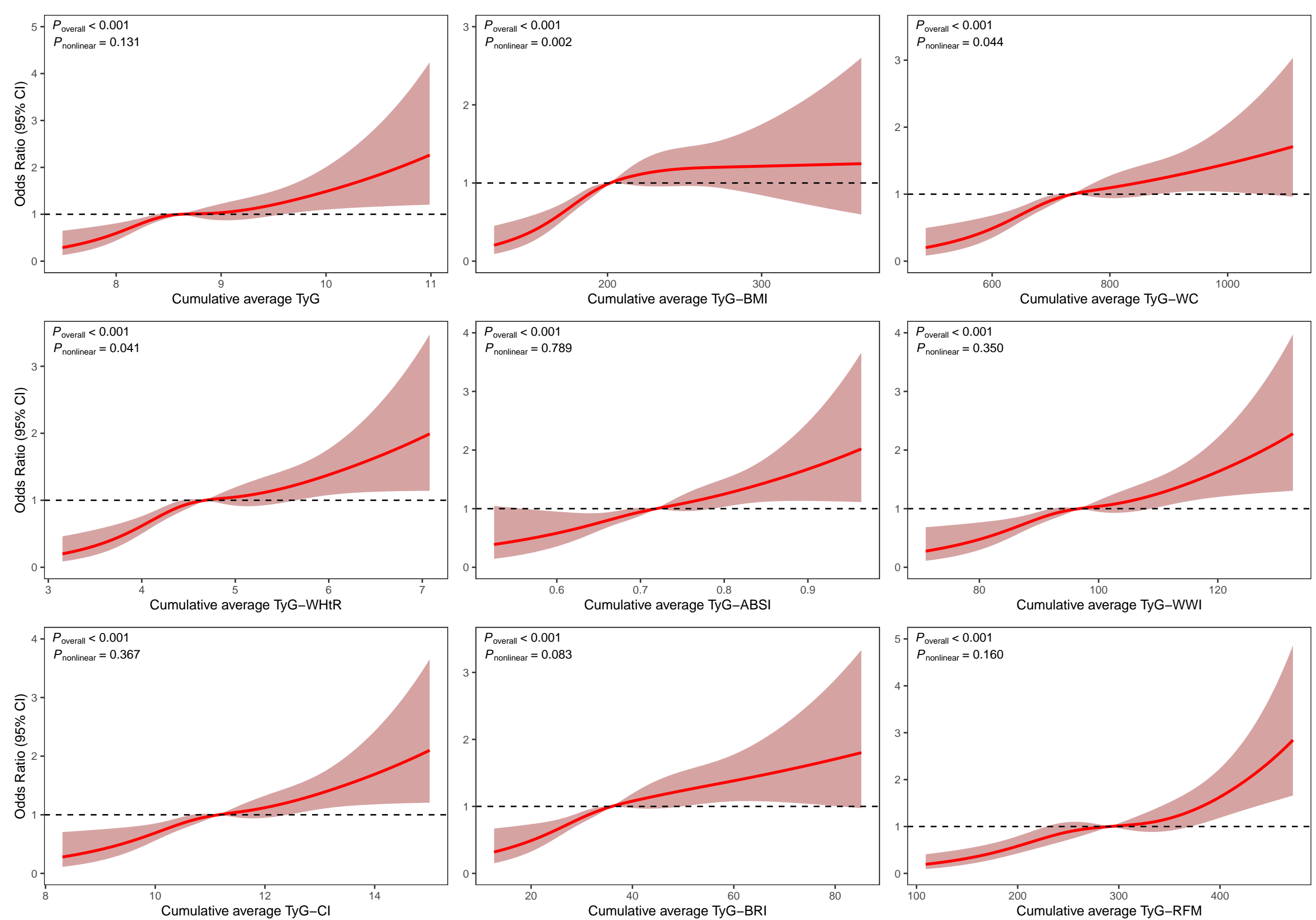

Supplement: Supplementary file 1 [file nutrients-17-02212-s001.zip › Figure S5.pdf]

A

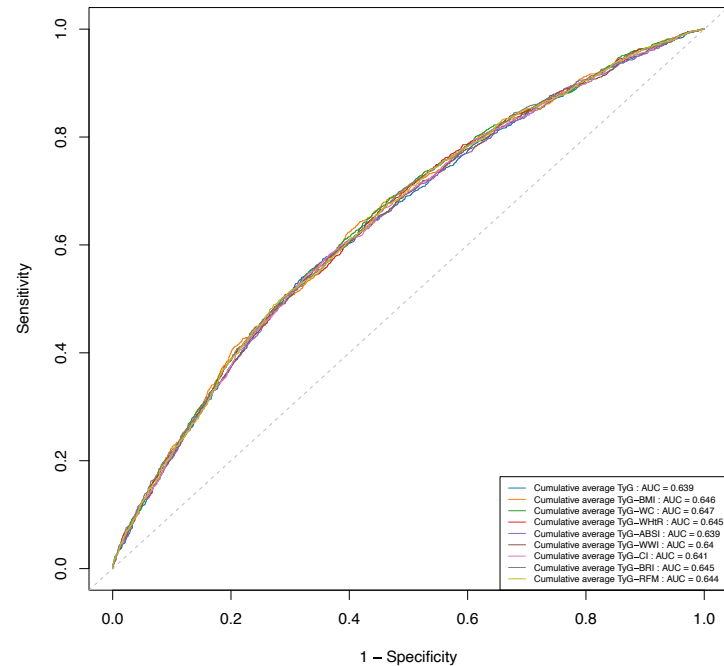

B

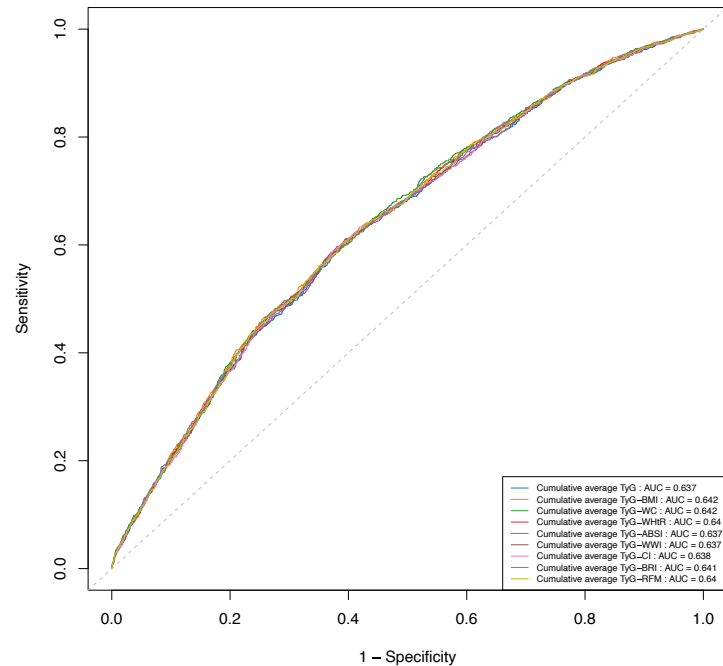

C

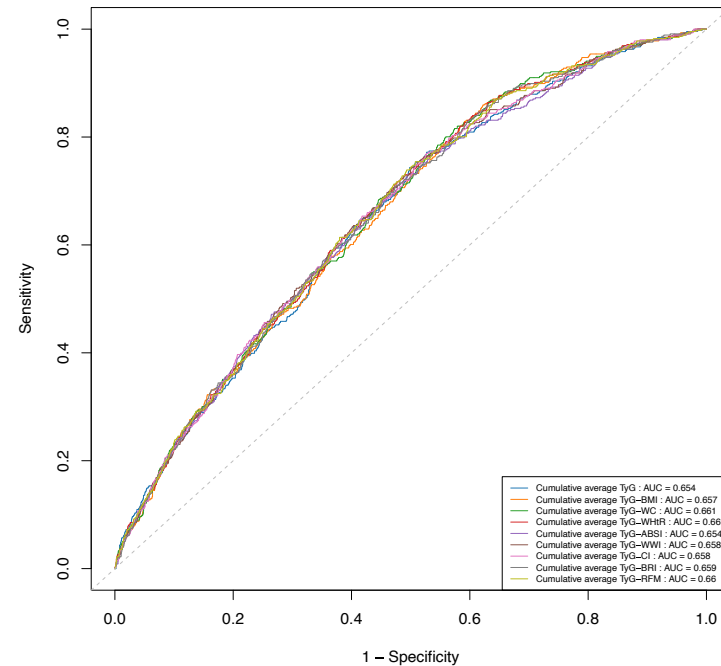

Supplement: Supplementary file 1 [file nutrients-17-02212-s001.zip › Figure S6.pdf]

# Heart disease

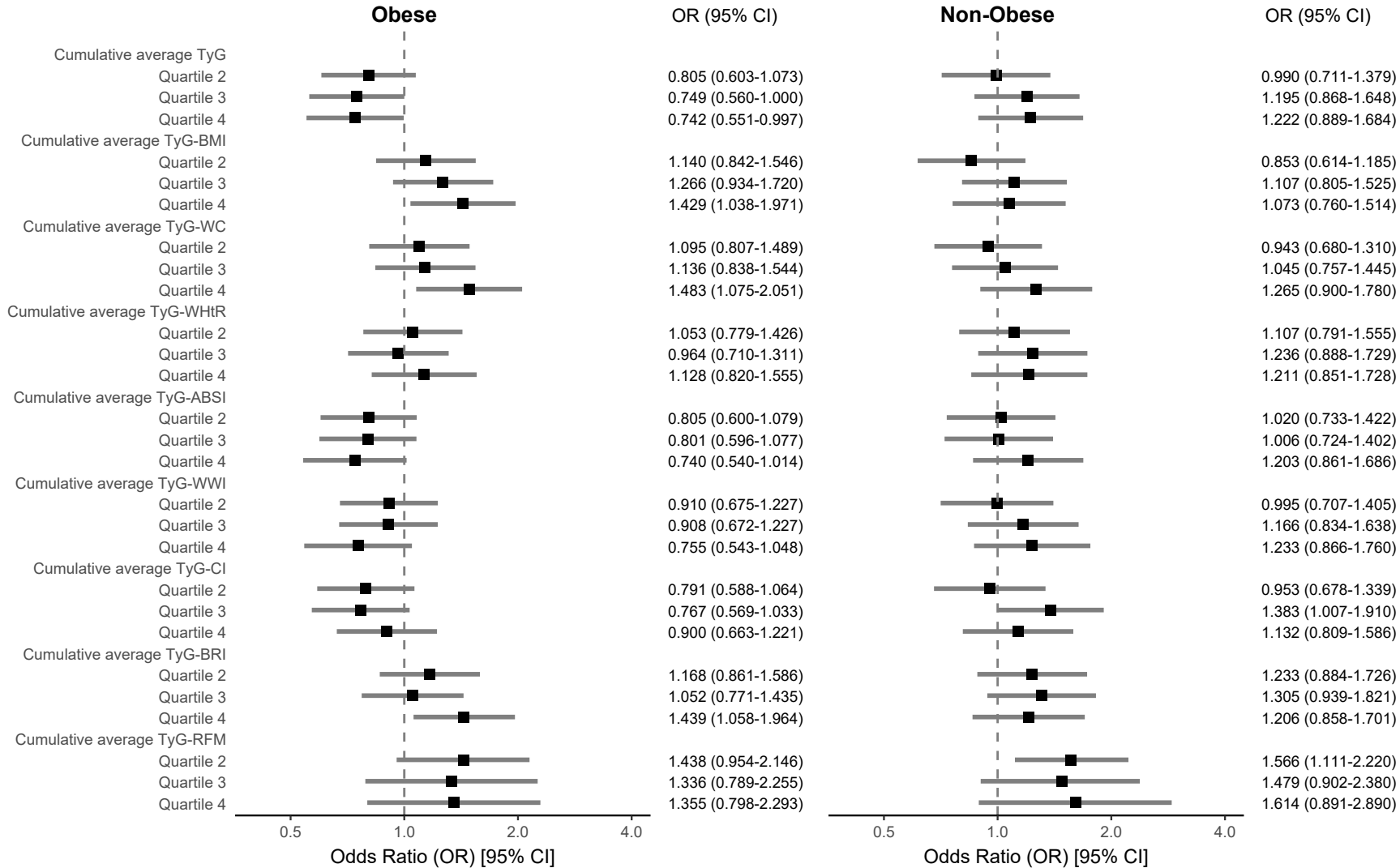

Supplement: Supplementary file 1 [file nutrients-17-02212-s001.zip › Figure S7.pdf]

# Heart disease

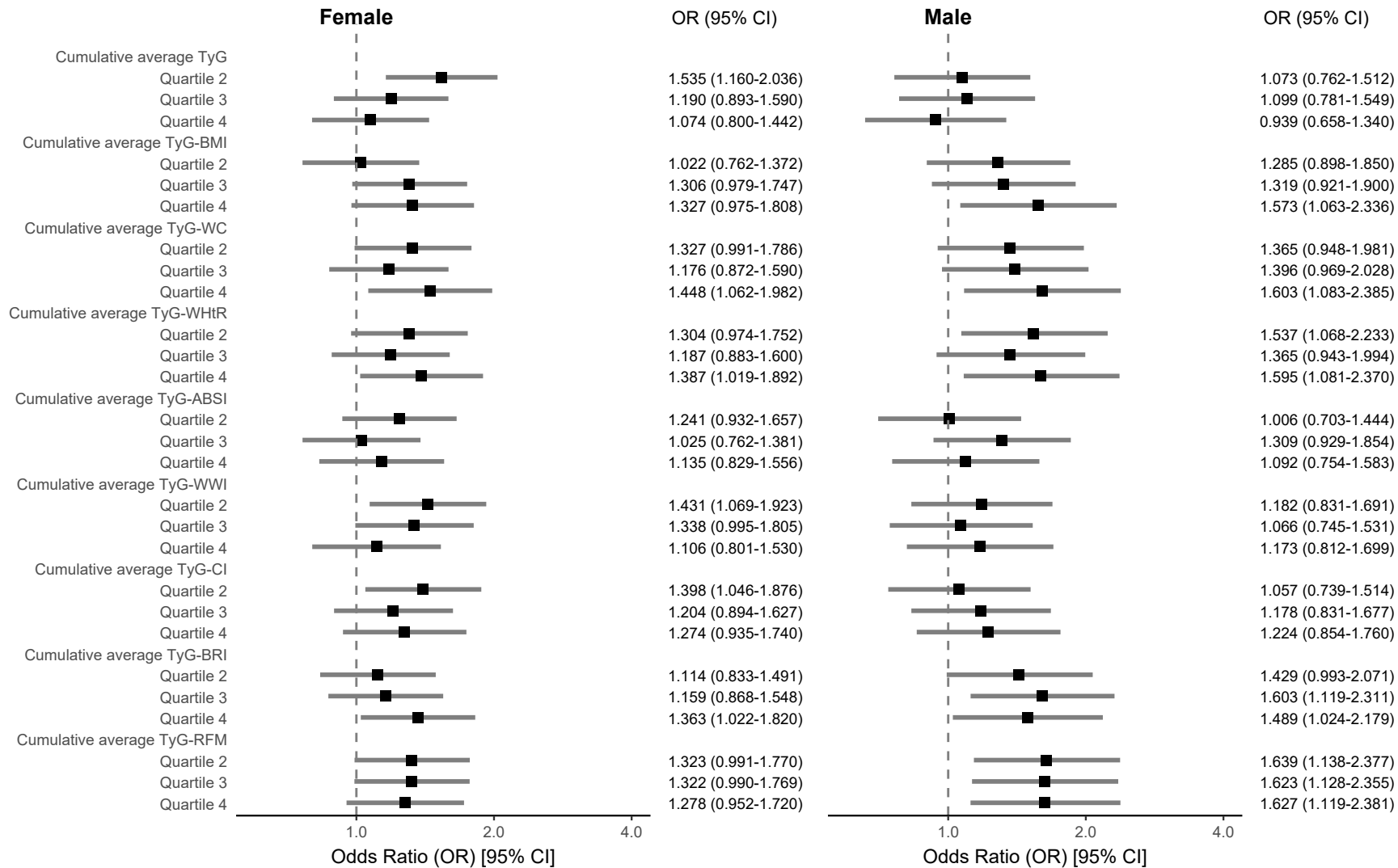

Supplement: Supplementary file 1 [file nutrients-17-02212-s001.zip › Figure S8.pdf]

# Heart disease

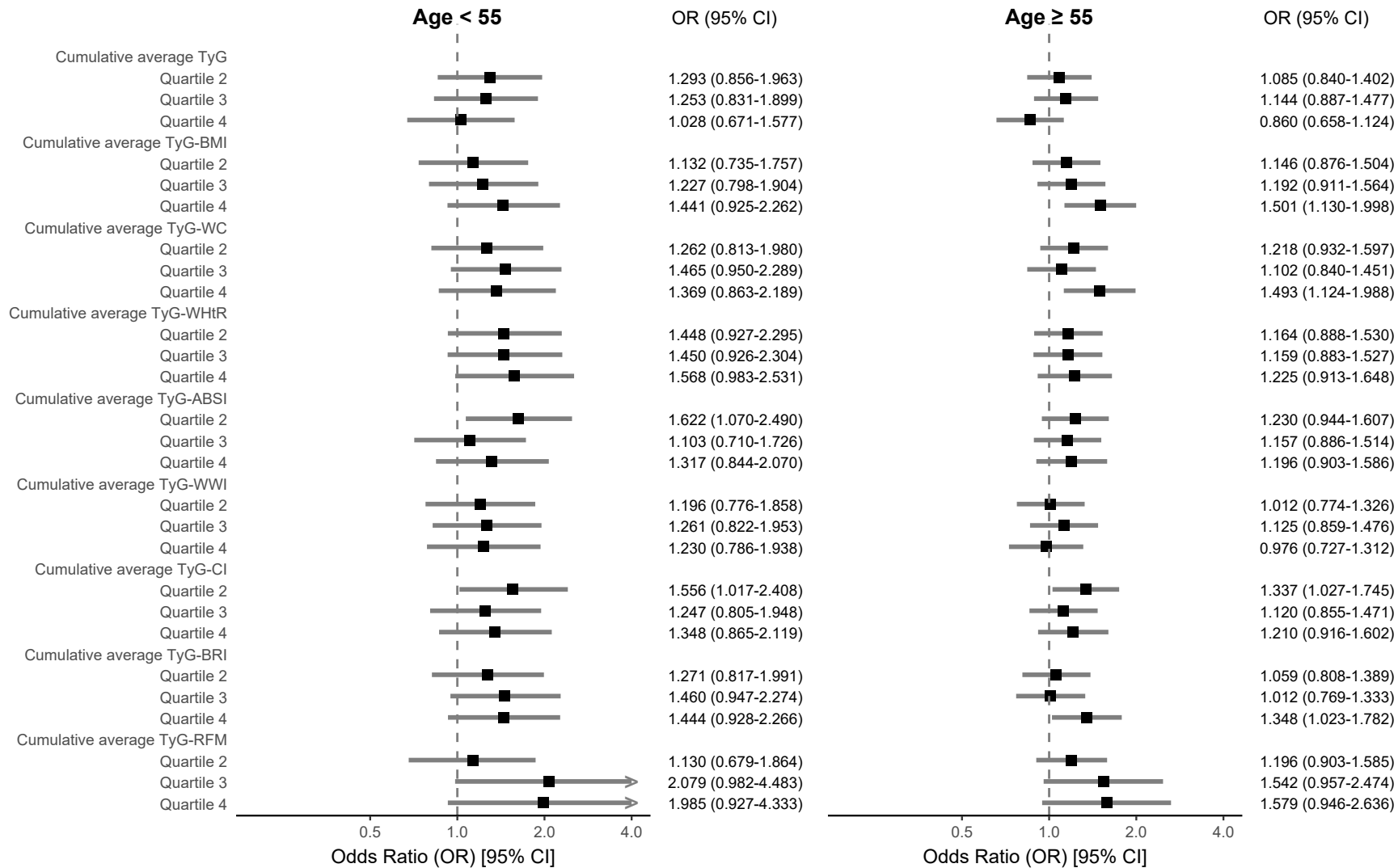

Supplement: Supplementary file 1 [file nutrients-17-02212-s001.zip › Figure S9.pdf]
